# New Materials Based on Cationic Porphyrins Conjugated to Chitosan or Titanium Dioxide: Synthesis, Characterization and Antimicrobial Efficacy

**DOI:** 10.3390/ijms20102522

**Published:** 2019-05-22

**Authors:** Kelly A. D. F. Castro, Nuno M. M. Moura, Flávio Figueira, Rosalina I. Ferreira, Mário M. Q. Simões, José A. S. Cavaleiro, M. Amparo F. Faustino, Armando J. D. Silvestre, Carmen S. R. Freire, João P. C. Tomé, Shirley Nakagaki, A. Almeida, M. Graça P. M. S. Neves

**Affiliations:** 1 QOPNA & LAQV-REQUIMTE, Department of Chemistry, University of Aveiro, 3810-193 Aveiro, Portugal; ffigueira@ua.pt (F.F); msimoes@ua.pt (M.M.Q.S.); jcavaleiro@ua.pt (J.A.S.C.); 2 CICECO, Departamento de Química, Universidade de Aveiro, 3810-193 Aveiro, Portugal; armsil@ua.pt (A.J.D.S.); cfreire@ua.pt (C.S.R.F.); 3 CESAM, Department of Biology, University of Aveiro, 3810-193 Aveiro, Portugal; rosalina.ferreira@ua.pt (R.I.F.); aalmeida@ua.pt (A.A.); 4 CQE, Departamento de Engenharia Química, Instituto Superior Técnico, Universidade de Lisboa, Av. Rovisco Pais, n1, 1049-001 Lisboa, Portugal; jtome@tecnico.ulisboa.pt; 5 Laboratório de Bioinorgânica e Catálise, Departamento de Química, Universidade Federal do Paraná, Curitiba, Paraná 81531-990, Brasil; shirleyn@ufpr.br

**Keywords:** Photosensitizers, cationic porphyrins, aPDT, immobilization, chitosan, TiO_2_

## Abstract

The post-functionalization of 5,10,15-tris(1-methylpyridinium-4-yl)-20-(pentafluorophenyl)porphyrin tri-iodide, known as a highly efficient photosensitizer (PS) for antimicrobial photodynamic therapy (aPDT), in the presence of 3- or 4-mercaptobenzoic acid, afforded two new tricationic porphyrins with adequate carboxylic pending groups to be immobilized on chitosan or titanium oxide. The structural characterization of the newly obtained materials confirmed the success of the porphyrin immobilization on the solid supports. The photophysical properties and the antimicrobial photodynamic efficacy of the non-immobilized porphyrins and of the new conjugates were evaluated. The results showed that the position of the carboxyl group in the mercapto units or the absence of these substituents in the porphyrin core could modulate the action of the photosensitizer towards the bioluminescent Gram-negative *Escherichia coli* bacterium. The antimicrobial activity was also influenced by the interaction between the photosensitizer and the type of support (chitosan or titanium dioxide). The new cationic porphyrins and some of the materials were shown to be very stable in PBS and effective in the photoinactivation of *E. coli* bacterium. The physicochemical properties of TiO_2_ allowed the interaction of the PS with its surface, increasing the absorption profile of TiO_2_, which enables the use of visible light, inactivating the bacteria more efficiently than the corresponding PS immobilized on chitosan.

## 1. Introduction

Nowadays, antimicrobial photodynamic therapy (aPDT) is considered to be a promising approach to inactivate a broad spectrum of pathogens, namely, microorganism strains with high resistance to conventional antibiotics [1,2,3]. The objectives and procedures behind aPDT are similar to those of photodynamic therapy (PDT), where the production of reactive oxygen species (ROS) provided by the activation of a photosensitizer (PS), at an adequate radiation wavelength, in the presence of molecular oxygen, is responsible for the oxidation of cellular components in target tissues, leading to cell death. The process can involve electron-transfer reactions from the PS triplet state to surrounding substrates, thereby facilitating cytotoxic species, such as superoxide anion, hydroxyl and lipid-derived radicals (mechanism Type I) and/or energy transfer from the PS triplet state to ground state molecular oxygen (^3^O_2_) to produce excited-state singlet oxygen (^1^O_2_) (mechanism Type II). Most of the success behind this approach is associated with the availability of photosensitizers with adequate structural features. In this regard, porphyrins are recognized as an essential class of derivatives to be associated with PDT and aPDT applications, since their action can be triggered under visible light [3,4,5,6,7,8,9,10,11,12,13].

Several reports found in literature show that Gram-positive and Gram-negative bacterial strains are susceptible to ROS produced by porphyrins; however, their efficacy is highly dependent on the used porphyrin structure and its charge. In particular, neutral, cationic or anionic porphyrin derivatives can inactivate Gram-positive bacteria [14], but an effective photo-inactivation of Gram-negative bacteria (without membrane disrupting agents) requires the presence of positive charges in the porphyrin structure [14,15,16,17,18,19,20]. The relationship between the porphyrin surrounding charges and the capability to inactivate Gram-negative bacteria can be explained by taking the bacteria physiology into consideration. While the Gram-positive bacteria cell wall is composed by a thick porous layer of peptidoglycan with attached teichoic acids, the Gram-negative bacteria cell wall includes a relatively thin layer of peptidoglycan surrounded by an asymmetric highly impermeable bilayer with an inner leaflet of phospholipids and outer leaflets of lipopolysaccharide. Each lipopolysaccharide imparts a strongly negative charge on the surface of Gram-negative bacterial cells, limiting the permeation of neutral and negatively charged PS [21]. However, PS containing positive charges are able to promote a strong electrostatic interaction with the negatively charged sites at the outer surface of the bacterial cells, thus facilitating their accumulation in key subcellular compartments.

The possibility of extending the aPDT protocol to clinical and environmental contexts, usually requires the PS encapsulation into delivery agents or its immobilization on solid supports [22,23]. Regarding the latter perspective, porphyrins have already proved to be an effective PS in the control of insect pests, food pathogenic microorganisms, and disinfection/sterilization of surfaces and residual waters [3,24,25,26,27]. For instance, porphyrins incorporated into chitosan films and nanomagnetic materials provided a high degree of reusability without affecting their aPDT capabilities [22,23,27,28,29,30]. The development of photoactive materials is also particularly relevant in the inactivation of bacteria able to form biofilms [28,31,32,33]. For instance, Kishen and co-workers [32] studied the efficacy of Rose Bengal conjugated with chitosan against dental biofilms and recently our group reported that porphyrin derivatives attached to chitosan are efficient materials for preventing *Listeria innocua* biofilms proliferation [28].

Herein, the development of different photoactive materials was achieved by pairing the adequate porphyrin structure to the materials where it will be supported, aiming to modulate and to fine-tune the type of application where porphyrins can be used. Materials such as chitosan and titanium oxide (TiO_2_) exhibit properties of technological interest, namely those related to antimicrobial activity. Chitosan shows low toxicity, good adsorption to mucous membranes, antibacterial properties and the capacity to promote covalent or non-covalent interaction with other molecules, making it a promising material for drug encapsulation [34,35]. On the other hand, TiO_2_ is a relatively low-cost material that is also non-toxic and highly photoactive while keeping its stability when irradiated with a suitable wavelength [22,36,37]. In fact, TiO_2_ produces ROS that can be used to kill microorganisms, facilitating the disinfection/inactivation of some pathogens under ultraviolet light irradiation [37,38]. Both materials show antimicrobial capabilities and, combined with porphyrins, it is possible to fine-tune and enhance those features [27,39,40].

In recent years, our studies have focused on the design of porphyrin derivatives with adequate structural features to be used as PS in aPDT, namely of Gram-negative and Gram-positive bacteria [7,14,20,22,28,41,42,43,44,45]. In this context, the development of photoactive functional materials, as well as their reusability, represents an important challenge for a sustainable development of aPDT protocols [22].

**Scheme 1:**
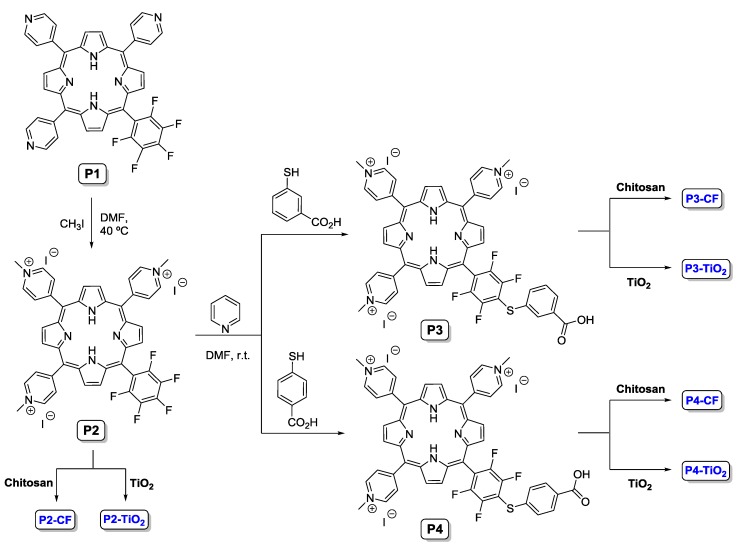
Synthesis of porphyrinic derivatives and their immobilization on chitosan and on TiO_2_.

During our studies concerning aPDT, we found that the tricationic porphyrin 5,10,15-tris(1-methylpyridinium-4-yl)-20-(pentafluorophenyl)porphyrin tri-iodide (**P2** in Scheme 1) bearing a pentafluorophenyl group is a highly efficient PS due to its ability to photoinactivate a wide range of microorganisms under a lower irradiation protocol than the one needed for other PS [46,47,48,49,50,51]. In an attempt to develop new materials based on that porphyrinic core and solid supports with antimicrobial capabilities such as chitosan and titanium dioxide, we decided to decorate the *meso*-pentafluorophenyl group of **P2** with other substituents containing carboxylic groups which will improve the interaction of the macrocycle with chitosan and titanium dioxide. It is expected that the synergistic combination of the **P2** derivatives with the antimicrobial capability of the materials could lead to an effective inactivation of both the Gram-positive and the Gram-negative bacteria upon irradiation with visible light.

In the present study, the further functionalization of the efficient photosensitizer **P2** with carboxylic groups was achieved by involving nucleophilic aromatic substitution of the *p*-fluorine atom of the C_6_F_5_ unit with 3- or 4-mercaptobenzoic acid (Scheme 1); it was considered that the position of the carboxylic group could influence the photoinactivation efficiency of the PS and/or of the PS hybrid materials. The new porphyrins obtained, **P3** and **P4**, both bearing carboxylic groups, and also (for comparison) the template **P2** were subsequently immobilized on chitosan films (**CF**) or on **TiO_2_** giving rise to materials **Px-CF** or **Px-TiO_2_**, respectively (where **x** stands for the porphyrin derivative number). The efficacy of the photodynamic action of **P2**, **P3** and **P4** and of the new materials was assessed in the presence of recombinant bioluminescent *E. coli*. This Gram-negative bacterium is an excellent model to monitor the effectiveness of a photoinactivation process, since its light output is a sensitive sign of its metabolic activity [16,19,20,52,53,54,55]. Additionally, *E. coli* is one of the most common Gram-negative pathogens in humans, causing several serious illnesses, and it is known to develop multidrug resistance [38,56].

## 2. Results and Discussion

### 2.1. Synthesis of Porphyrin Derivatives

The general synthetic procedure used to obtain the new cationic porphyrin derivatives **P3** and **P4** bearing carboxyl groups is provided in Scheme 1. Both porphyrins were obtained using the tricationic porphyrin **P2**. The neutral precursor **P1** was prepared by reacting pyrrole with the adequate stoichiometric proportion of pentafluorobenzaldehyde and pyridine-4-carbaldehyde in a mixture of acetic acid/nitrobenzene according to a literature procedure [46]. The quaternization of the pyridyl groups was carried out with methyl iodide and the required cationic porphyrin **P2** [5,10,15-tris(1-methylpyridinium-4-yl)-20-(pentafluorophenyl)porphyrin tri-iodide] was quantitatively obtained [46]. The following aromatic nucleophilic substitution took place with 3- or 4-mercaptobenzoic acid in the presence of pyridine and DMF at room temperature. The required porphyrins **P3** and **P4** have been obtained with 90% and 93% yield, respectively [28,57,58]. The structures of the obtained products were confirmed by spectroscopic techniques (Figures S1–S15). The analytical data obtained for the intermediates is in accordance with the data previously published [59].

The ^1^H NMR spectra of compounds **P3** and **P4** showed the expected signals assigned to their structures. The insertion of 3-mercaptobenzoic acid residue rendered a non-symmetric pending group giving rise to three signals depicted at δ 7.69, δ 8.05–7.97 and δ 8.23 ppm, assigned to the resonances of H5, H4 and H6, and H2 of the 3-mercaptobenzoic unit. On the other hand, the insertion of the 4-mercaptobenzoic group gave rise to two doublets depicted at δ 8.03 and δ 7.78 ppm. All these assignments were confirmed through correlations found in 2D NMR spectra (COSY). The ^19^F NMR spectra of both derivatives also confirmed the success of the nucleophilic substitution by the disappearance of the resonance signal of the *p*-fluorine nucleus.

The absorption, emission and excitation spectra of **P3** and **P4** were recorded in DMF solutions (1 μM) at 298 K and were compared with those from the precursors **P1** and **P2** (see Table 1 and Figures S13 and S14, Supporting Information). The absorption spectra of **P3** and **P4** show the typical profile expected for free base *meso*-tetraarylporphyrins with the Soret band at 420 nm and four weak Q bands in the 500–650 nm range. The phyllo type Q-bands (IV > II > III > I) evidence a significant reduction in the intensity of bands III and I, which is a typical feature of tetraarylporphyrins containing electronegative substituents at the *ortho* positions of the phenyl rings [60].

The steady-state fluorescence emission spectra of all compounds, after their excitation at ca. 420 nm, show essentially two bands: one centered at ca. 648 nm and the other at 711 nm (Table 1 and Figure S16). These emission bands can be assigned to Q_x_(0–0) and Q_x_(0–1) transitions, typical of the free-base porphyrins with a D_2h_ symmetry due to a nearly unchanged vibronic state upon excitation [61]. The perfect match between the absorption and the excitation spectra observed for all the compounds rules out the presence of any emissive impurities (Figures S14 and S17, Supporting Information).

The Stokes shift for **P3** and **P4** suggest a change in the electronic nature of the excited state compared to the ground state [62]. The fluorescence yields (Φ_F_) were determined using Equation (1), and 5,10,15,20-tetraphenylporphyrin (**H_2_TPP)** as reference (0.11 in DMF) [63,64]. In this equation, Φ_F_ is the emission quantum yield of the sample, Φ_st_ the emission quantum yield of the standard, A_st_ represents the absorbance of the standard and A_s_ the absorbance of the sample at the excitation wavelength. Furthermore, S_st_ and S_s_ represent the integrated emission band areas of the standard **H_2_TPP** and of the sample, respectively. The results summarized in Table 1 show that **P1** and **P3** presented a slightly higher fluorescence quantum yield than **P2** and **P4**.

(1) $$\Phi_{F}=\Phi_{st}\left(\frac{S_{s}}{S_{st}}\;\times \frac{A_{st}}{A_{s}}\right)$$

### 2.2. Immobilization of Porphyrins on Chitosan

The immobilization of the cationic porphyrins **P2**, **P3** and **P4** on chitosan was performed by adding the adequate amount of each porphyrin dissolved in THF to a solution of chitosan in an aqueous acetic acid solution (1% *v*/*v*), according to a procedure previously reported by our group [28]. The amount of porphyrin immobilized was calculated indirectly by UV-Vis, considering the amount of unreacted porphyrin in the supernatants resulting from the washing process. The chitosan-porphyrin films, designated as **P2-CF**, **P3-CF** and **P4-CF**, were prepared by dissolving the porphyrins and chitosan in a 1% acetic acid solution followed by casting in a ventilated oven at 30 °C. The percentage of each porphyrin immobilized on the chitosan support is summarized in Table 2.

In Table 2 it is possible to observe that **P3** and **P4** lead to higher immobilization percentages than **P2,** most certainly due to the strong interactions (electrostatic and/or hydrogen bond) between the porphyrinic carboxylic unit and the chitosan cationic ammonium groups. On the other hand, although **P2** interacts with chitosan in a lesser extent, this interaction seems also to be facilitated by hydrogen bonding involving the fluorine atoms and the chitosan OH and NH_2_ groups; additionally, one cannot exclude the interactions between the π-conjugated electron cloud of the macrocycle rings and the hydroxyl groups of chitosan [65].

All the chitosan-porphyrin films were characterized using a wide array of solid-state spectroscopic techniques such as UV-Vis and fluorescence, ATR-FTIR and powder X-ray diffraction (PXRD).

**Figure 1:**
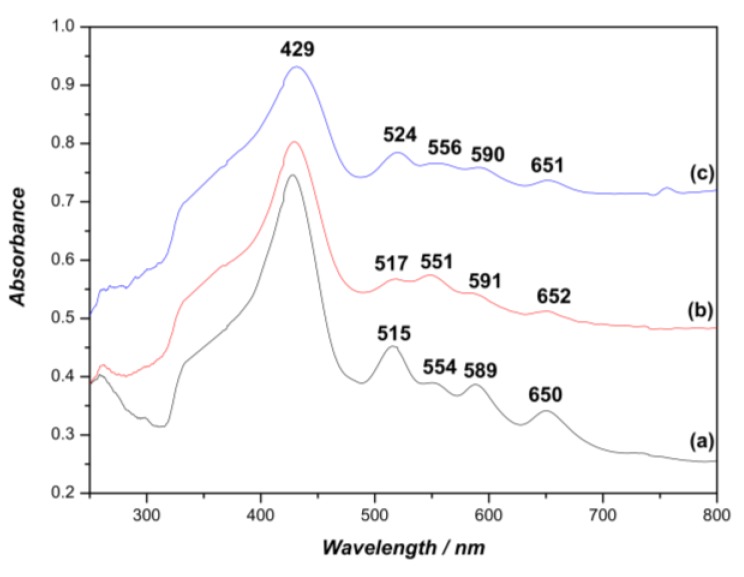
Solid state UV-Vis spectra of (a) **P2-CF**, (b) **P3-CF** and (c) **P4-CF**.

The presence of the porphyrin on the chitosan films **P2-CF, P3-CF** and **P4-CF** was promptly confirmed through UV-Vis and fluorescence spectra in the solid state. All the absorption spectra (Figure 1) displayed the typical Soret band accompanied by four Q-bands. All these bands are red-shifted and broader when compared with the corresponding absorption bands in solution (Table 1) [66]. A similar situation was observed with the UV-Vis of **P2**, **P3** and **P4** in the solid state (Figure S15, Supporting Information). The Soret bands suffered a red-shift of ca. 10 nm and in the Q-band region, depending on the porphyrin, red-shifts of ca. 5–16 nm were also detected. These shifts are attributed to small alterations of porphyrin molecular environment due to its interaction with chitosan [28,67].

**Figure 2:**
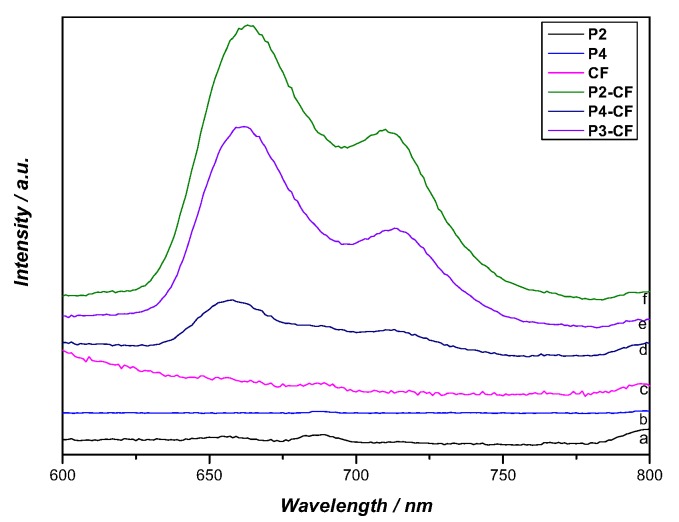
Emission spectra in the solid state for (a) **P2**, (b) **P3**, (c) **CF**, (d) **P4-CF**, (e) **P3-CF** and (f) **P2-CF**.

**Figure 3:**
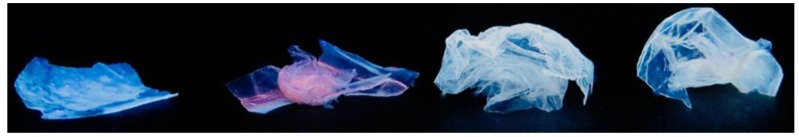
Digital photographs of **PS-CF**. From left to right: **CF**, **P2-CF**, **P3-CF** and **P4-CF** under ultraviolet light.

The solid-state fluorescence spectra of **P2, P3, P4** (Figure 2a–c) and of the immobilized porphyrins on chitosan (**P2-CF**, **P3-CF** and **P4-CF**, Figure 2e,f) are illustrated in Figure 2. All the hybrids show two emission bands in the red region upon excitation at 420 nm, which correspond to the S_1_→S_0_ transitions of the porphyrins. The two bands were observed at 663 and 711 nm for **P2-CF**, 661 and 714 nm for **P3-CF** and 657 and 712 nm for **P4-CF**. When compared with the corresponding emission of porphyrins **P2-P4** in solution (Table 1) a red-shift of ca. 9-15 nm was observed in the first emission band. The low emission observed in the solid state for **P2-P4** can probably be attributed to their tendency to form aggregates, leading to fluorescence quenching. Figure 3 shows the visual appearance of chitosan and of the **P2-CF**, **P3-CF** and **P4-CF** materials under ultraviolet light. The fluorescence intensities have not a linear dependence with the concentration of the porphyrin present in chitosan (**P3** and **P4** lead to higher immobilization percentages than **P2**, Table 2) but are dependent on the substituents present on the porphyrin core. This behavior is probably due to self-absorption and self-quenching of the PS supported in the films as described previously by Tian and co-workers [61].

The ATR-FTIR spectra of the **Px-CF** conjugates displayed the characteristic bands of chitosan (Figure S18, Supporting Information). The band at 3450 cm^−1^ is attributed to overlapped OH and N–H vibrations, while the bands at 2916 cm^−1^, 2867 cm^−1^ are associated with C–H and –C=O of the amide group CONHR vibrations of chitosan.

The bands at 1589, 1412 and 1332 cm^−1^ are associated with the bending vibrations of the N–H, C–H, O–H bonds; the vibration at 1152 cm^−1^ is due to ν_as_(C=O) oxygen bridges and the band at 893 cm^−1^ to ω(C–H) from the polysaccharide’s structure. Most of the characteristic peaks of the porphyrin core are significantly reduced, when compared with chitosan, due to the low porphyrin immobilization on **CF** films (Table 2).

The PXRD of **P3-CF** and **P4-CF** showed some differences in the crystallinity when compared with **CF**. The peak observed at 42.9° for **CF** due to the crystalline phase of chitosan was not observed in **P3-CF** and **P4-CF**, indicating that the porphyrin immobilization led to a loss of crystallinity [68]. More specifically, it is possible to note for **P3-CF** a new set of peaks at 8.5, 11.8 and 15.2°, attributed to the chitosan chain rearrangements during the porphyrin immobilization process. Most of the peaks in the PXRD of these materials are characteristic peaks of chitosan while the presence of peaks related to the supported porphyrins are not visible [28,69]. Interestingly, the PXRD of **P2-CF** shows a predominant amorphous phase of chitosan (Figure S20, Supporting Information).

### 2.3. Immobilization of Porphyrins on Titanium Dioxide

The immobilization of the cationic porphyrins **P2**, **P3** and **P4** on **TiO_2_** was performed by adding an adequate amount of each porphyrin dissolved in methanol to solid **TiO_2_** (see details in the experimental section).

The amount of each porphyrin immobilized on **TiO_2_** was also calculated by UV-Vis, considering the amount of porphyrin in the supernatants resulting from the washing process. The immobilization percentages of each porphyrin on the **TiO_2_** support are summarized in Table 3. It is possible to note that the presence of the carboxylic groups in the porphyrin core is a fundamental requisite for an efficient electrostatic interaction with the **TiO_2_** solid. Porphyrin **P2**, without the acidic groups, was completely removed after the washing process, while **P3** and **P4** showed higher than 90% immobilizations.

**Figure 4:**
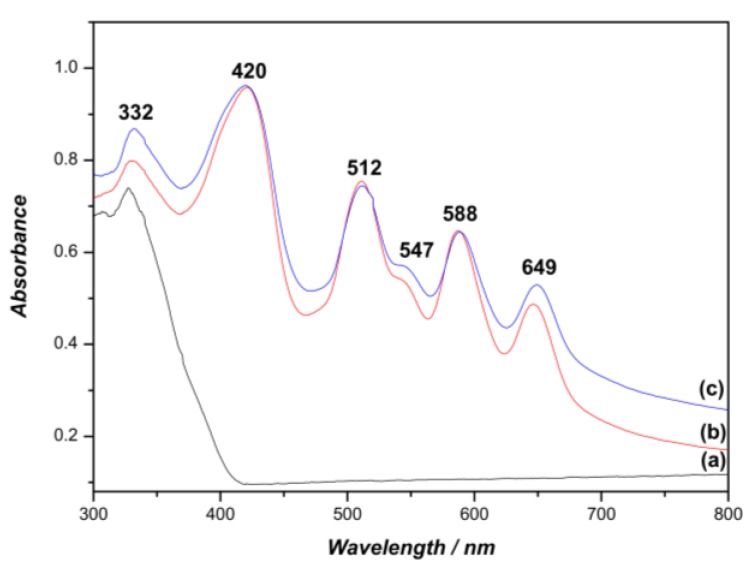
UV-Vis spectra in the solid state of (a) **TiO_2_**, (b) **P3-TiO_2_** and (c) **P4-TiO_2_**.

The UV-Vis spectra of the solids **P3-TiO_2_** and **P4-TiO_2_** show the typical porphyrin absorption bands confirming the success of **P3** and **P4** immobilization on **TiO_2_** (Figure 4). The wavelengths of the Soret band at 420 nm and the Q-bands at 512, 547, 588, 649 nm are similar to those of the non-immobilized porphyrins in solution. However, when the spectra are compared with the absorption spectra of the porphyrins in the solid state, a blue shift can be observed (Figure S15, Supporting Information) [70,71]. This is probably due to interactions between the porphyrin plane and the support in an attempt of the macrocycle to acquire a more planar conformation in order to maximize the electrostatic interaction [72].

**Figure 5:**
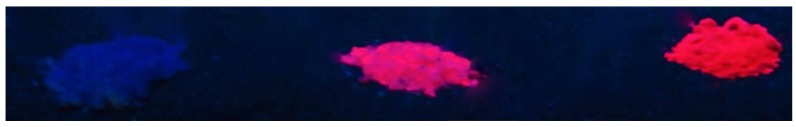
Digital photographs of **PS-TiO_2_**. From left to right: **TiO_2_**, **P3-TiO_2_** and **P4-TiO_2_** under UV light.

**Figure 6:**
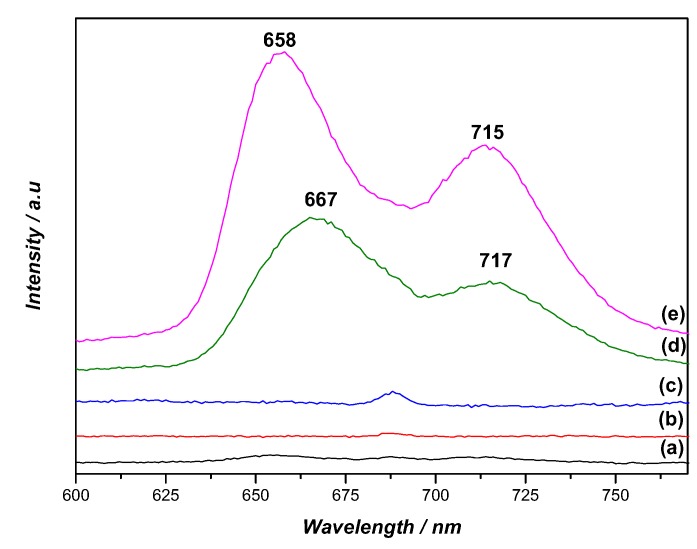
Emission spectra in the solid state of (a) **P3**, (b) **P4**, (c) **TiO_2_**, (d) **P4-TiO_2_**, and (e) **P3-TiO_2_**.

Figure 5 shows the solids **TiO_2_**, **P3-TiO_2_** and **P4-TiO_2_** under ultraviolet light and Figure 6 shows the corresponding emission spectra in the solid state. Contrarily to the low emission observed with **P3** and **P4** in the solid state, both hybrids showed a strong fluorescence. Hybrid **P3-TiO_2_** shows two emission bands at 658 and 715 nm, with the first one more pronounced than the second one (Figure 6e), while **P4-TiO_2_** exhibits the same profile but with the two bands at 667 and 717 nm (Figure 6d). The fluorescence excitation spectra (λ_em_ at 715 or 717 nm) show the typical features of porphyrins. These emission bands are slightly shifted when compared with those obtained when the same porphyrins were immobilized on chitosan. This behavior can be attributed to the distinct immobilization modes on the support and agrees with the distortions in the structure of the porphyrins indicated by the UV-Vis results. García-Sánchez and co-workers [73] observed a shift in the emission spectra for porphyrins immobilized on silica. The authors attributed this behavior to the different polarity existing inside the silica pores and the interactions with the surface groups.

The ATR-FTIR of **P3-TiO_2_** and **P4-TiO_2_** exhibit the typical vibration bands of titanium dioxide in the region of 3400 cm^−1^ (ν OH), at 1630 cm^−1^ attributed to the bending vibration of the hydroxyl group on the surface of **TiO_2_** and at 481 cm^−1^ corresponding to the vibration of the Ti-O-Ti bonds [74]. In these solids, some characteristic bands due to the presence of the porphyrins were also observed at approximately 1593 and 1408 cm^−1^ arising from the symmetric and asymmetric vibrations of the C=O group and at 1472 cm^−1^ due to C=C vibrations (Figure S19, Supporting Information).

**P3-TiO_2_** and **P4-TiO_2_** hybrids displayed a PXRD pattern similar to those obtained from the pure support (Figure S21, Supporting Information). The two intense peaks at 25.3° and 27.4° (2θ) attributed to the anatase (101) and rutile (110) phases of **TiO_2_**, can be clearly observed in the hybrids, showing that the porphyrins do not interfere with the **TiO_2_** crystallinity structure.

### 2.4. Singlet Oxygen Generation and Photostability

Knowing that the ability of a porphyrin or a material to act as a photosensitizer is related with its ability to generate ROS, namely ^1^O_2_, and also with its stability, these essential features were also considered in the characterization of the new compounds and materials.

**Figure 7:**
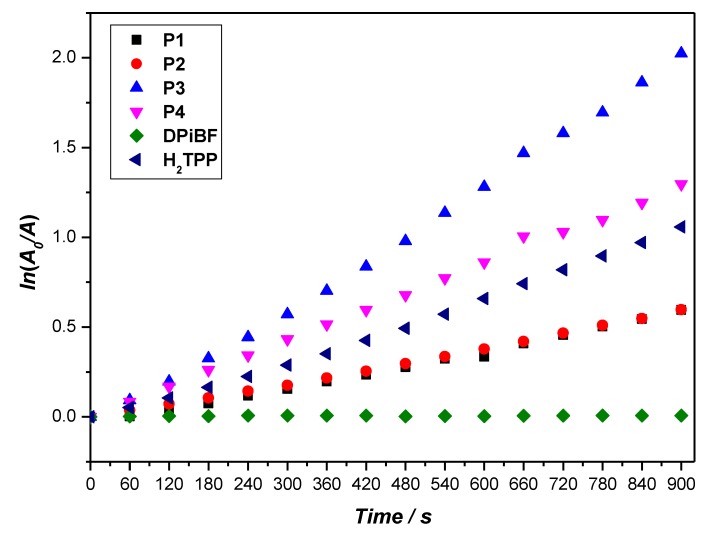
Time-dependent photodecomposition of DPiBF (50 µM) photosensitized by **P1**, **P2**, **P3** and **P4** in DMF upon irradiation with red light (640 ± 20 nm) with or without PS (0.5 µM). **H_2_TPP** was used as the reference.

The efficacy of compounds **P3** and **P4** to generate singlet oxygen was verified using 1,3-diphenylisobenzofuran (DPiBF) as a scavenger of singlet oxygen (^1^O_2_), and further compared with the ability of the precursors **P1** and **P2** (Figure 7). The degradation of DPiBF was monitored by the decrease of its band at 415 nm, after its irradiation in the presence of each porphyrin and atmospheric oxygen. The reaction between ^1^O_2_ and DPiBF in a 4+2 process affords 1,2-dibenzoylbenzene giving an indirect evidence of ^1^O_2_ generation.

The results summarized in Figure 7 show that all the porphyrins are good generators of ^1^O_2_, but the best efficacy was found for the new porphyrins **P3** and **P4**. These two new porphyrins can generate ^1^O_2_ more efficiently than **H_2_TPP**, also considered an excellent ^1^O_2_ generator [75]. The best ^1^O_2_ generator was **P3**, followed by **P4** and **H_2_TPP**, and finally by **P1** and **P2**, both with a similar efficacy. These results suggest that the presence of the acid group and its position are important features for the efficiency in ^1^O_2_ generation and the lower efficacy of **P1** and **P2** is probably associated with aggregation phenomena.

**Figure 8:**
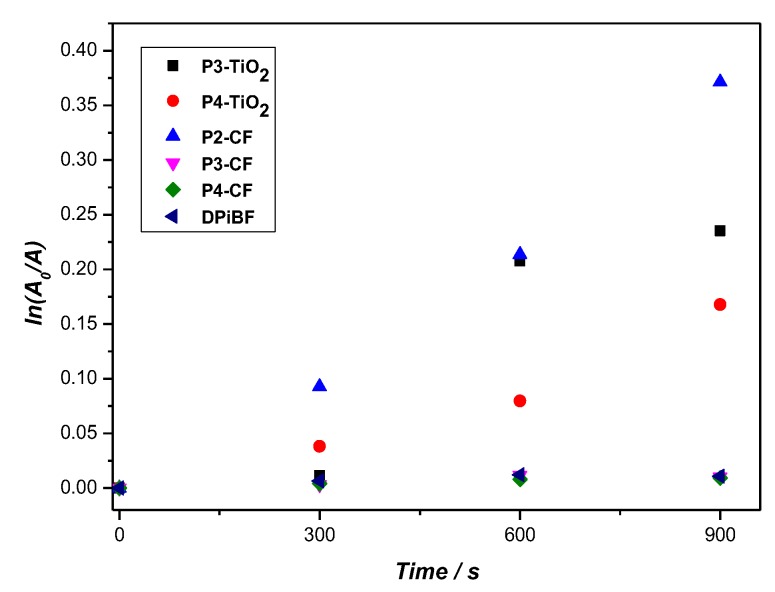
Time-dependent photodecomposition of DPiBF (50 µM) photosensitized by **P2-CF**, **P3-CF**, **P4-CF**, **P3-TiO_2_** and **P4-TiO_2_** in DMF upon irradiation with red light (640 ± 20 nm) using 2.0 µM of PS.

The DPiBF decomposition provided by the materials **PS-CF** and **PS-TiO_2_** was also investigated and the results are summarized in Figure 8. The results show that **P3-TiO_2_** is a more efficient ^1^O_2_ producer than **P4-TiO_2_**. This result is in line with the better performance of **P3** to generate ^1^O_2_ when compared with **P4**. Considering the chitosan materials, the higher DPiBF decay was caused by **P2-CF** and none of the other materials (**P3-CF** and **P4-CF**) were able to generate ^1^O_2_ under the tested conditions.

Interestingly, **P2-CF** showed almost the same ^1^O_2_ production as **P2** in solution. This behavior might be related with an aggregation attenuation or an increased photostability of the porphyrin after its immobilization on the chitosan support.

The photostability studies were performed with **P2-P4** in PBS, monitoring the decrease in absorbance of the corresponding Soret bands after different times of irradiation with a LED array at an irradiance of 10 mW·cm^−2^. **P4** suffered a decay of 64%, followed by **P2** (41%) and **P3** (22%) during the irradiation period (Figure S22). Based on the observed degradation profiles, the studies were repeated in the absence of light, to verify if the decrease was not associated with an aggregation phenomenon. In fact, **P4** showed a decrease of 41%, **P2** (28%) and **P3** (22%) in dark conditions, showing that somewhere up to a 23% decrease, the decrease is related to the photodecomposition of the macrocycle. In sum, **P2** and **P4** are more susceptible to photodegradation upon illumination, while **P3** was quite photostable over the irradiation period investigated (30 min).

### 2.5. Photoinactivation Studies

It is commonly accepted that the success of photoinactivation closely depends on the structure of the photosensitizing agent [14]. As previously stated, the charge of the PS is extremely important for the inactivation of the microbial agents and, generally, positively charged PS are more efficient for Gram-negative strains, even if they are at low concentrations when compared to neutral or negatively charged PS [52]. Studies with cationic PS demonstrate that these derivatives are more likely to inactivate both Gram-positive and Gram-negative bacteria without the help of membrane disrupting agents [47,76].

**Figure 9:**
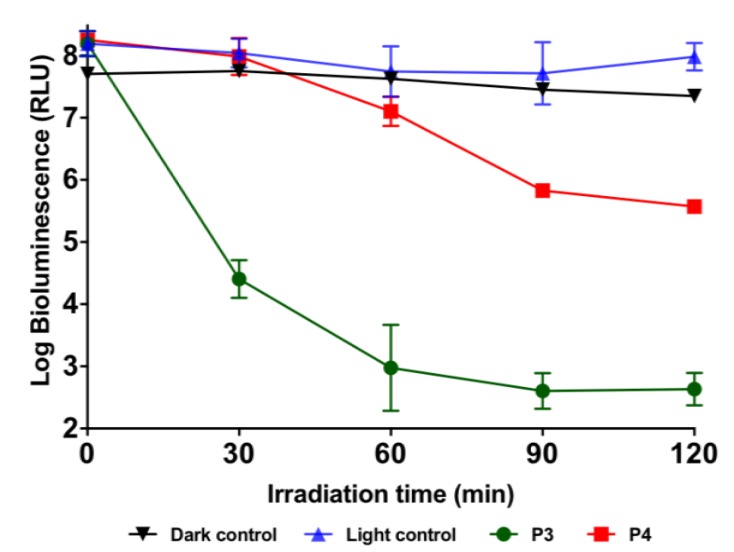
Photoinactivation of bioluminescent *E. coli* in the presence of **P3-P4** at the concentration of 5.0 μM under 3.0 mW cm^−2^ of white light irradiation. The values represent the mean of three independent experiments with three replicates and the error bars indicate the standard deviation.

The biological assays started by evaluating the photodynamic action of the new cationic porphyrins **P3** and **P4** at the concentration of 5.0 µM, under white light towards the recombinant bioluminescent *E. coli* (Figure 9). As mentioned above, this Gram-negative bacterium is an excellent bacterial model to monitor the effectiveness of a photoinactivation process, since its light output is a highly sensitive reporter of its metabolic activity. These set of experiments were not performed in the presence of the non-immobilized **P2** since its efficacy has already been studied by some of us [46,47].

The results summarized in Figure 9 show that the bioluminescence of *E. coli* was not affected by light in the absence of the PS (light control) neither by the direct effect of any of the tested PS in the absence of light (dark control). These results indicate that any variation in the bioluminescence was due to the photodynamic effect of the PS. Photosensitizers **P3** and **P4** showed significant differences in terms of inactivation efficiency (ANOVA–*p* < 0.05) (Figure 9). **P3** at a concentration of 5.0 µM was more effective than **P4**, causing a reduction of approximately 3.6 log in the bioluminescence, after only 30 min of irradiation. At this concentration, **P3** inactivated completely *E. coli* (decrease of the bioluminescent signal below the quantification limit, 2 log) after 120 min of irradiation (with a total light dose of 21.6 J·cm^−2^) whilst **P4** caused an inactivation of 3 log after 120 min of irradiation. Compound **P3** can be considered a good PS against *E. coli*, since it showed a similar efficiency as **P2** towards *E. coli* at the same concentration (5.0 μM) and under the same light irradiance [47]. The difference observed for each PS can be justified by a balance between the efficiency to produce ^1^O_2_ (**P2** ~ **P3** > **P4**) and their affinity to the bacteria which is more dependent on the PS structure.

Using different approaches (e.g., scanning electron microscopy (SEM), transmission electron microscopy (TEM), spectrophotometry, ^1^D electrophoresis, atomic force microscopy, proteomic, lipidomic, infrared spectroscopy) to evaluate the ultrastructural, morphological and functional changes in bacterial cells, at initial stages and during the course of the PDI process, it seems that the most affected cellular constituents in *E.coli* are external targets, namely proteins and lipids, which can affect normal cell membrane functionality or lead to significant cell membrane disintegration [77,78,79,80].

**Figure 10:**
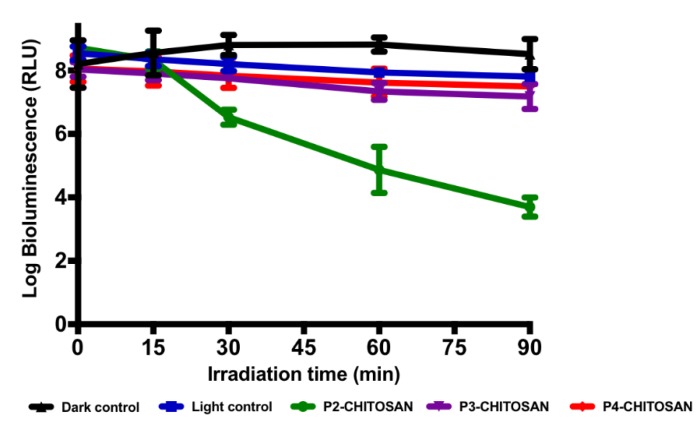
Photoinactivation of the bioluminescent *E. coli* in the presence of porphyrins **P2**, **P3** and **P4** supported on chitosan at the concentration of 3.0 µM under 3.0 mW cm^−2^ of white light irradiation. The values represent the mean of three independent experiments with three replicates and the error bars indicate the standard deviation.

**Figure 11:**
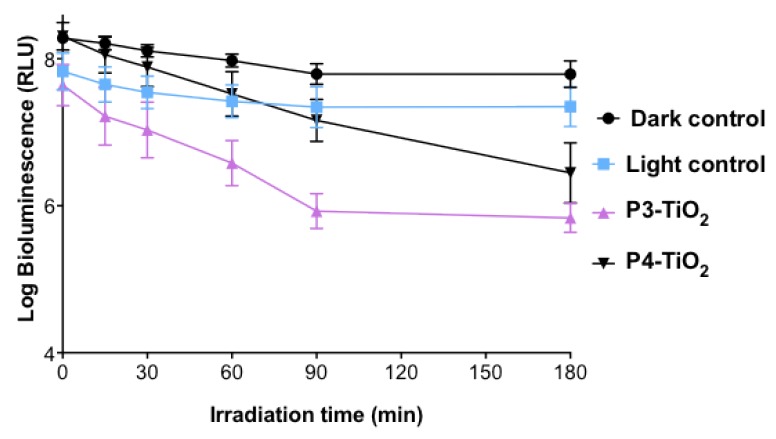
Photoinactivation of bioluminescent *E. coli* with 10 μM of the porphyrins **P3** and **P4** supported on **TiO_2_** under 3.0 mW·cm^−2^ of white light irradiation. The values represent the mean of three independent experiments with three replicates and the error bars indicate the standard deviation.

The results obtained for the photoinactivation efficacy of the new conjugates towards the bioluminescent *E. coli* were evaluated and are compiled in Figure 10 (**Px-CF**) and Figure 11 (**Px-TiO_2_**). It is known that chitosan can act as an antimicrobial agent, although Gram-negative bacteria are less susceptible to its effects [81].

Figure 10 shows for **P2-CF** and after 90 min of irradiation, a noticeable decrease of the bioluminescent signal to approximately 4.1 log. However, no significant differences were detected in the bioluminescent signal when the bacteria were exposed to **P3** and **P4** supported on chitosan (decrease of 0.63 and 0.31 log, respectively).

These results are most certainly related to the inability of these porphyrins to produce oxygen singlet, after being immobilized in chitosan. The porphyrins photodynamic performance in solution follows the order **P2** ~ **P3** > **P4**; however, when supported on chitosan films, only **P2-CF**, with the capacity to generate oxygen singlet, has a significant effect on the bacterial cells. Studies show that chitosan might have either bactericidal or bacteriostatic properties, depending on the type of bacterium, which is related to the thickness of the cell wall. Recent data shows that chitosan tends to act as bacteriostatic rather than bactericidal, which can also explain the visible changes in the dark controls [82].

The positive charges of chitosan and **P2** can interfere with the negatively charged *E. coli* cell envelope, resulting in alterations of membrane permeability, which in combination with the ^1^O_2_ justifies the efficiency observed for **P2-CF**.

The combination of the 5,10,15,20-tetrakis(4-sulphonatophenyl)porphyrin (**TPPS^4−^**) or 5,10-bis(4-methylphenyl)-15,20-bis(4-*N*,*N*,*N*-trimethylammoniumphenyl)porphyrin (**MPAP^2+^**) and chitosan produced a potentiated photodynamic inactivation (PDI) effect. Both porphyrins **TPPS^4−^** and **MPAP^2+^** at 5.0 µM in the presence of 0.25 mg/mL of chitosan caused a reduction of 3.5 log in the concentration of viable cells after a total light dose of 5.4 J·cm^−^^2^ [81].

As shown in Figure 10, neither **P3** nor **P4** immobilized on chitosan are good photosensitizers. **P3** and **P4**, when combined with chitosan, show almost no effect on the cells. Durantini and co-workers showed that chitosan was toxic to *E. coli* by itself at high concentrations (0.75 or 1.00 mg/mL) [81]. The concentration used in this work was 0.34 mg/mL and, as expected, no cytotoxic effect was observed for *E. coli* in the dark. Similar results were observed for **P2-CF** at a concentration of 3.0 μM after a total light dose of 10.8 J·cm^−2^. The porphyrins 5,10,15,20-tetrakis(4-aminophenyl)porphyrin (***p*-TAPP**) and **TMPyP** incorporated into chitosan showed a considerable bactericidal effect under irradiation at 590 nm (2 log reduction after 120 min for ***p*-TAPP** and 4 log reduction after 140 min for **TMPyP**) [40]. The best results were obtained when **P2** was incorporated into chitosan. The similarities seen in the UV-Vis spectra before and after the PDI biological assays show that **PS-CF** materials were photostable (Figure S23).

The positive effect of chitosan was also indicated when Luksiene and co-workers studied the photodynamic inactivation of the Gram (−) food pathogen *Salmonella enterica* using the chlorophyllin–chitosan hybrid as PS [30]. The authors verified that the inactivation caused by chlorophyllin (1.8 log, at 15 μM, light dose 38 J·cm^−2^) and chitosan (*ca* 1.8 log) separately gave rise in the presence of photoactivated **Chl–CHS** complex to a remarkable and swift decrease of *Salmonella enterica* viability (7 log) [30]. Considering that the inactivation of *E. coli* was achieved at lower PS concentration (3.0 μM vs 15 μM) and light dose (16.2 vs 38 J·cm^−2^), we can conclude that the new hybrid **P2-CF** is also a very promising material. No cytotoxic effect observed for chitosan alone can be justified to the low concentrations used since high concentrations (0.75 or 1.00 mg/mL) are required for *E. coli* inactivation, as showed by Durantini [81].

As stated before, **TiO_2_** can be used as PS for the inactivation of some pathogens under ultraviolet light irradiation. The electronic properties of **TiO_2_** can be easily modulated by linking a PS to its surface and the immobilization of the PS induces an extension of the absorption profile of **TiO_2_** enabling the use of the visible light [83]. Macyk and co-workers showed that a **TiO_2_** suspension did not result in any significant *E. coli* photoinactivation under visible light [84]. When **P3** and **P4** are supported on TiO_2_, it is noticeable that **P3** and **P4** at a concentration of 10 μM cause a reduction of approximately 2 log in the *E. Coli* bioluminescence after 180 min (32.4 J/cm^2^) (Figure 11). **P3-TiO_2_** and **P4-TiO_2_** have a higher effect on the bacterial cells compared to **P3-CF** and **P4-CF**. Compared to **PS-TiO_2_**, the ^1^O_2_ production from these **PS-CF** was much less efficient and could be disregarded.

TiO_2_-graphene functionalized with 5,10,15,20-tetrakis(4-carboxyphenyl)porphyrin showed a decrease of 64% in the concentration of viable cells of *E. coli* after 440 min under light irradiation (450 W xenon lamp) [27]. In this study, both porphyrins **P3** and **P4** supported on **TiO_2_** showed a decrease of 99% in the concentration of viable cells of *E. coli* after 180 min.

Conversely, higher amounts of **PS-TiO_2_** did not increase the photodynamic effect. In fact, the increase of material as a powder in these tests can obstruct light from reaching the PS, hampering the photosensitization process.

## 3. Materials and Methods 

All analytical grade chemicals used in this study were purchased from Sigma-Aldrich or Merck. Chitosan was purified following the procedure described in the literature [28]. **H_2_TPP** was synthesized as described in the literature [85]. **TiO_2_** was Evonik-Degussa P25.

^1^H, ^19^F and ^13^C solution NMR spectra were recorded on a Bruker Avance 300 (300.13 and 282.38 MHz, respectively), 500 (125.76 MHz for ^13^C) spectrometer. DMSO-d_6_ was used as solvent and tetramethylsilane (TMS) as the internal reference; the chemical shifts are expressed in ppm and the coupling constants (*J*) in Hertz (Hz). Unequivocal ^1^H assignments were made using 2D COSY (^1^H/^1^H). Electronic spectra (UV-Vis) were obtained on a Shimadzu UV-2501PC spectrophotometer, in the 350–800 nm range. Spectra of the solid samples were recorded on a Jasco V-560 spectrophotometer with reflectance accessory (JASCO ISV-469) in absorbance mode in the 250–850 range. Attenuated Total Reflectance Transmission Fourier Transform Infrared (ATR-FTIR) spectra were registered on a FTIR Bruker Tensor 27 spectrophotometer with ATR accessory ATR (ATR Golden Gate Diamond, Specac) in the 350–4000 cm^−1^ range; the spectra were collected with a resolution of 4 cm^−1^ and accumulation of 256 scans. For the X-ray diffraction (PXRD) measurements, self-oriented solids were placed on neutral glass sample holders. The measurements were performed in the reflection mode using a Panalytical Empyrean diffractometer (Cu Kα_1,2_ X-radiation, λ_1_ =1.540598 Å and λ_2_ =1.544426 Å), equipped with an X’Celerator detector and a flat-plate sample holder in a Bragg- Brentano para-focusing optics configuration (40 kV, 50 mA). Intensity data were collected by the step counting method (step 0.02) in continuous mode. The fluorescence emission spectra were recorded in DMF in 1 × 1 cm quartz optical cells under normal atmospheric conditions on a computer-controlled Horiba Jobin Yvon FluoroMax-3 spectrofluorimeter. The widths of both excitation and emission slits were set at 2.0 nm. The fluorescence quantum yield (φ_F_) of porphyrins was calculated in DMF by comparison of the area below the corrected emission spectra using **H_2_TPP** as standard (λ_exc_ at 420 nm, φ_F_ = 0.11 in DMF) [64]. Fluorescence spectra of the solid samples were recorded using a fiber optic system connected to a Horiba-Jobin Yvon FluoroMax-3 spectrofluorimeter for appropriately λ (nm) of the solid compounds.

### 3.1. Synthesis of Porphyrin Derivatives

Porphyrin **P1** was obtained by the reaction of the appropriate aldehydes in acetic acid and nitrobenzene, according to a literature procedure (see Scheme 1) [59]. The purity was confirmed by TLC and by ^1^H and ^19^F NMR spectroscopy.

**Porphyrin P2.** To a solution of **P1** (0.106 mmol, 0.075 g) in *N,N*-dimethylformamide (DMF; 1 mL), an excess of methyl iodide (0.2 mL) was added in a sealed tube. The mixture was stirred overnight at 40 C. After cooling, diethyl ether was added to precipitate the porphyrin. The solid was isolated by filtration and washed with diethyl ether. The desired porphyrin was crystallized from CH_2_Cl_2_:CH_3_OH/hexane to yield **P2**. After that, the solid was dried under vacuum and then characterized.

**P2**: Yield: 98%; ^1^H NMR (300 MHz, DMSO-d_6_): *δ* 9.63–9.46 (8H, m, H-β), 8.92 (6H, d*, J =* 9.1 Hz, Ar-Py-*m*-H), 8.42 (6H, d*, J =* 9.1 Hz, Ar-Py-*o*-H), 4.32 (9H, s, -CH_3_), −3.15 (2H, s, *N*-H) ppm. ^19^F NMR (280 MHz, DMSO-d_6_): *δ* −135.45 (2F, dd, *J =* 24.8 and 6.1 Hz, Ar-*o*-F), −150.17 (1F, t, *J =* 22.6 Hz, Ar-*p*-F), between -160.17 and −160.37 (2F, m, Ar-*m*-F) ppm. UV-Vis (DMF) λ_max_, nm (log ε): 420 (4.90), 510 (3.83), 544 (3.19), 584 (3.36), 640 (2.70).

**Porphyrin P3.** To 3-mercaptobenzoic acid (0.015 mmol, 0.002 g) dissolved in DMF (5.0 mL) and containing 0.5 mL of pyridine, porphyrin **P2** (0.014 mmol, 0.015 g) was added after 30 min in a single portion. The reaction mixture was stirred for 1 h at ambient temperature; afterwards, diethyl ether was added to precipitate the porphyrin, the solid was filtered and washed with diethyl ether. Porphyrin **P3** was obtained by crystallization from methanol/chloroform.

**P3:** Yield: 90%; ^1^H NMR (300 MHz, DMSO-d_6_): *δ* 9.50–9.47 (8H, m, Ar-Py-*m*-H and H-*β*), 9.17 (6H, brs, H-*β*), 9.02 (6H, dd, *J* *=* 11.1, 6.7 Hz, Ar-Py-*o*-H), 8.23 (1H, s, S-*Ar*-COOH), 8.05–7.97 (2H, m, S-*Ar*-COOH), 7.69 (1H, t, *J* = 7.7 Hz, S-*Ar*-COOH), 4.72 (9H, s, -C*H*_3_), −3.11 (s, 2H, *N*-H) ppm. ^19^F NMR (280 MHz, DMSO-d_6_) *δ* −129.69 (2F, dd, *J =* 26.9 and 11.5 Hz, Ar-*o*-F), −134.52 (2F, dd, *J =* 26.9 and 11.5 Hz, Ar-*m*-F) ppm. ^13^C NMR (125 MHz, DMSO-d_6_): *δ* 166.6 (S-Ar-COOH), 162.4 (ArPy-Por, CH), 156.3 (ArPy-Por, CH), 144.3 (Ar-Py-*m*-H, -N-CH), 133.7 (Ar-Py-*o*-H, -N-CH), 133.3 (S-Ar, CH), 132.3 (S-Ar, CH), 132.2 (S-Ar, CH), 130.4 (S-Ar, CH), 129.9 (S-Ar, CH), 128.8 (S-Ar, CH), 116.2 (Por-ArF_4,_ C-Por), 115.9, 103.9 (C-ArF_4,_ C-S), 48.0 (CH_3_) ppm. UV-Vis (DMF) λ_max_, nm (log ε): 420 (5.03), 512 (3.92), 546 (3.41), 584 (3.48), 640 (2.90). HRMS-ESI(+): calcd. for C_51_H_36_F_4_N_7_O_2_S [M]^+·^ 886.2571 found 886.2555.

**Porphyrin P4.** 4-Mercaptobenzoic acid (0.03 mmol, 0.004 g) was dissolved in DMF containing 0.5 mL of pyridine. The reaction mixture was kept under stirring at ambient temperature for 30 min. Afterwards, porphyrin **P2** (0.03 mmol, 0.031 g) was added, and the reaction mixture was stirred for 1 h at ambient temperature. Then, diethyl ether was added to precipitate the porphyrin, the solid was filtered and washed with diethyl ether. The solid was dissolved in methanol and, after concentration, it was re-precipitated by the addition of acetone.

**P4:** Yield: 93%; ^1^H NMR (300 MHz, DMSO-d_6_): *δ* 9.57–9.50 (8H, m, Ar-Py-*m*-H and H-β), 9.18 (6H, brs, H-β), 9.06–9.00 (6H, m, Ar-Py-*o*-H), 8.03 (2H, d, *J =* 8.2 Hz, S-*Ar*-COOH), 7.78 (2H, d, *J =* 8.2 Hz, S-*Ar*-COOH), 4.73 (9H, s, –C*H*_3_), −3.10 (2H, s, N-*H*) ppm. ^19^F NMR (280 MHz, DMSO-d_6_): *δ* −129.31 (2F, dd, *J* = 26.7, 11.3 Hz, Ar-*o*-F), −134.42 (2F, dd, *J* = 26.7, 11.3 Hz, Ar-*m*-F) ppm. ^13^C NMR (125 MHz, DMSO-d_6_): *δ* 167.2 (S-Ar-COOH), 156.3 (ArPy-Por, CH), 144.3 (Ar-Py-*m*-H, -N-CH), 132.3, 132.2 (Ar-Py-*o*-H, -N-CH), 130.5 (S-Ar, CH), 127.7 (S-Ar, CH), 116.3 (Por-ArF_4,_ C-Por), 103.9 (C-ArF_4,_ C-S), 48.0 (CH_3_) ppm. UV-Vis (DMF) λ_max,_ nm (log ε): 420 (5.29), 510 (4.15), 544 (3.61), 584 (3.73), 640 (3.01). HRMS-ESI(+): calcd. for C_51_H_36_F_4_N_7_O_2_S [M]^+·^ 886.2571 found 886.2578.

### 3.2. Immobilization of Porphyrins on Chitosan

The porphyrin-chitosan (Px-chitosan where x = 2, 3 or 4) solids was prepared in two steps: 

(i) 250 mg of chitosan were dissolved in 7.0 mL of 1% (*v*:*v*) aqueous acetic acid. Afterwards, porphyrins **P2**, **P3** or **P4** (5.0 mg) were added and the mixtures were stirred for 24 h at 30 °C. The solvent was evaporated under reduced pressure and the resulting solids were filtered and exhaustively washed with water and DMF. The solutions resulting from the washing process were analyzed by UV-Vis in order to quantify the Px that leaked from the chitosan and the loading of the porphyrin in each solid was calculated. The solids were dried in the oven at 55 °C for 72 h.

(ii) Each sample obtained (about 100 mg) was dissolved in 10 mL of 1% (*v*:*v*) aqueous acetic acid. The suspension was maintained at 40 °C under stirring until complete solubilization of the solid (3 h). The solution was poured into molds (5.0 × 5.0 cm) and allowed to dry at room temperature for 72 h. The films were denominated as **P2-CF**, **P3-CF** and **P4-CF** and characterized by ATR-FTIR, UV-Vis, PXRD and fluorescence of solid samples.

### 3.3. Immobilization of Porphyrins on Titanium Dioxide

To a solution of **P2**, **P3**, or **P4** in methanol (0.789 µM, 10 mL) 0.5 g of **TiO_2_** were added. The resulting suspension was stirred at ambient temperature for 6 h. The solids were isolated by centrifugation, washed with methanol and dried in the oven at 55 °C for 24 h. UV-Vis analysis of the solutions resulting from the washing process was performed and the loading of the porphyrin in each solid was calculated. The solids were named as **P3-TiO_2_** and **P4-TiO_2_** and characterized by the techniques described above. **P2** was completely removed after the washing process of **P2-TiO_2_**, so this hybrid was not obtained.

### 3.4. Singlet Oxygen Generation and Photostability

In a 1 cm x 1 cm quartz cell, and to a solution of DPiBF at 50 μM, 0.5 µM of each PS in DMF were added and irradiated with a LED array at an irradiance of 10 mW cm^−2^. The LED array is composed of a matrix of 5 × 5 LEDs making a total of 25 light sources with an emission peak centered at 640 nm and a bandwidth at half maximum of ± 20 nm. Irradiation was conducted at ambient temperature under stirring. The decreasing of DPiBF was monitored by UV-Vis at 415 nm at intervals of 1 min during 15 min. The absorption decay is proportional to the production of ^1^O_2_. For the **Px-CF** and **Px-TiO_2_** materials, a similar protocol was adopted, but the PS concentration used was 2.0 µM and the monitorization by UV-Vis was performed after centrifugation of the suspension irradiated and only the times 0, 5, 10 and 15 min were registered. Controls were also included. The experiments were performed in duplicate. **H_2_TPP**, an efficient singlet oxygen generator, was included as a reference.

The photostability studies of the porphyrin derivatives were performed by irradiation of a solution of each porphyrin in DMF (5.0 µM) with a LED array. The solutions were stirred and kept at ambient temperature. The stability of each porphyrin derivative was verified by UV-Vis at regular intervals up to 60 min.

### 3.5. Bacterial Strain and Culture Growth Conditions

The efficiency of the porphyrins in solution and after being incorporated on the materials to photoinactivate a bioluminescent *E. coli* was evaluated using a reliable and quick method that allows the monitorization of the activity of this bacterial strain in real time. Bioluminescent *E. coli* was transformed as described in a previous work [46] and stored at −80 °C in 10% glycerol. For the transformation, the plasmids pHK724 and pHK555 were inserted into competent cells of *E. coli* Top 10 (Invitrogen, USA), resulting in a bioluminescent strain. These plasmids contain the luxoperon from the bioluminescent marine bacterium *Vibrio fischeri*, required to produce light without the addition of exogenous substrates [46]. Before each assay, an aliquot of bioluminescent bacteria stored at −80 °C in 10% glycerol was aseptically plated on tryptic soy agar (TSA, Merck) supplemented with 50 mg·mL^−1^ of ampicillin (Amp) and with 34 mg·mL^−1^ of chloramphenicol (Cm), and stored at 4 °C. Before each assay, one isolated colony was aseptically transferred to 10 mL of tryptic soy broth (TSB, Merck) medium previously supplemented with Amp and Cm and was grown overnight at 25 °C under stirring (120 rpm). Afterwards, an aliquot was transferred into 10 mL TSB under the same growth conditions to reach stationary growth phase. An optical density at 600 nm (OD_600_) of 1.6 ± 0.1 corresponded to ≈10^8^ colony forming units (CFU) mL^−1^ [46]. 

### 3.6. Irradiation Conditions for Photosensitization

All samples were exposed, in parallel, to white light (PAR radiation, 13 OSRAM 21 lamps of 18 W each, 380 700 nm) with a fluence rate of 3.0 mW cm^−2^ (measured with a light meter LI-COR model LI-250, Li-Cor Inc., Lincoln, NE, USA), at ambient temperature of less than 25 °C under 100 rpm mechanical stirring.

### 3.7. Photosensitization Procedure

Bacterial cultures mentioned above were grown overnight and then tenfold diluted in phosphate buffered saline (PBS), at pH 7.4, so that ~10^7^ relative light units (RLUs) were reached. Five milliliters of the bacterial suspension were evenly distributed in glass beakers (previously sterilized) and the appropriate volume of the PS was added in order to attain final concentrations of 5.0 µM. For the immobilized PS, the corresponding mass of **PS-CF** or **PS-TiO_2_** was added in order to achieve the final concentration of 3.0 or 10 µM, respectively. For the non-immobilized PS, the suspensions were incubated for 15 min in the dark to allow the binding of the PS to the bacterial cells, and subsequently exposed to irradiation conditions, during a maximum period of 120 min. For the materials prepared, the dark incubation time was 60 min and after which they were exposed to the same irradiation conditions, during a maximum period of 90 min and 180 min for **Px-CF** and **Px-TiO_2_**, respectively.

Dark and light controls were included in the experiments. In the light control, the bacterial suspensions without PS were exposed to the same irradiation protocol as the test suspensions. In the dark control, the PS was added and protected from light with aluminum foil. At the initial time, and after established times of irradiation, the bioluminescence was measured in the luminometer (GloMax^®^ 20/20 Luminometer, Promega, Madinson, WI, USA). The percentage of decay of the bioluminescence is proportional to the concentration of viable cells (CFU). Three independent experiments with three replicates were performed and the results were averaged.

### 3.8. Statistical Analysis

Statistical analysis was performed in GraphPadPrism 7.0. The significance of the PDI effect of each PS and of the irradiation time on bacterial cell viability was assessed by an unvaried analysis of variance (ANOVA) model with the Bonferroni post hoc test. Normal distributions were assessed by the Bartlett’s test and homogeneity of variances was assessed by the Brown-Forsythe test. A value of *p* < 0.05 was considered significant.

## 4. Conclusions

We successfully accomplished the chemical modification of the 5,10,15-tris(1-methylpyridinium-4-yl)-20-(pentafluorophenyl)porphyrin. Moreover, two cationic porphyrins were obtained as a result of the substitution of the *p*-fluorine atoms on the *meso*-phenyl with thio-carboxylate acids. These cationic porphyrins were successfully immobilized on two different supports: chitosan and titanium oxide. The efficiency of the aPDT process against *E. coli* seems dependent on the structure of the porphyrin and on its ability to produce singlet oxygen. The results showed that the choice of the support and of the adequate porphyrin can be modulated to improve the efficiency of aPDT. The electronic properties of TiO_2_ allow linking the PS to its surface, increasing the absorption profile of TiO_2_, which enabled the use of the visible light, inactivating bacteria more efficiently than the **PS-CF**. 

## Figures and Tables

**Table 1:** Selected photophysical properties of porphyrins **P1–P4** in DMF.

Porphyrin	Soret Band λ_max_ (nm)	Q Bands λ_max_ (nm)				λ_emission_ (nm) ^a)^	Stokes Shift (nm)	Φ_F_^b)^
**P1**	420	512	545	585	641	648/711	7	0.03
**P2**	420	510	544	584	640	649/711	9	0.01
**P3**	420	512	546	584	640	649/711	9	0.03
**P4**	420	510	544	584	640	648/711	8	0.01

^a)^ The optical density of all the samples was 0.05 at 420 nm; ^b)^ using **H_2_TPP** as the reference in DMF (Φ_F_ = 0.11) [64].

**Table 2:** Porphyrin immobilization in chitosan.

Solid	Loading (mol·g^−1^) ^a)^	*w*/*w* (%) ^b)^	Immobilization (%) ^c)^
**P2-CF**	7.87 × 10^−6^	0.89	44%
**P3-CF**	1.03 × 10^−6^	1.30	65%
**P4-CF**	1.09 × 10^−6^	1.38	69%

^a)^ Porphyrin (mol) / mass (g) of solid prepared; ^b)^ mass (g) of porphyrin per gram of chitosan; ^c)^ immobilization percentage based on the relative mass of porphyrin used.

**Table 3:** Porphyrin immobilization on titanium dioxide solid results.

Solid	Loading (Porphyrin Per Mass of Solid Prepared) (mol g^−^^1^) ^a)^	*w*/*w*^b)^ (%)	Immobilization ^c)^ (%)
**P2-TiO_2_**	0 ^d)^	0^c^	-
**P3-TiO_2_**	1.44 × 10^−5^	1.82	91%
**P4-TiO_2_**	1.55 × 10^−5^	1.96	96%

^a)^ Porphyrin (mol) / mass (g) of titanium dioxide; ^b)^ mass (g) of porphyrin per mass (gram) of titanium dioxide; ^c)^ immobilization percentage based on the mass of porphyrin used; ^d)^ there was no interaction.

## Supplementary Materials

Supplementary materials can be found at https://www.mdpi.com/1422-0067/20/10/2522/s1.
