# Supplementary material for: New Materials Based on Cationic Porphyrins Conjugated to Chitosan or Titanium Dioxide: Synthesis, Characterization and Antimicrobial Efficacy
Source: Int J Mol Sci. 2019 May 22;20(10):2522. doi: 10.3390/ijms20102522 (PMC6566955; doi:10.3390/ijms20102522)
Supplement: Supplementary file 1 [file ijms-20-02522-s001.pdf]

Supporting Information

New materials based on cationic porphyrins conjugated to chitosan or titanium dioxide: synthesis, characterization and antimicrobial efficacy

Kelly A. D. F. Castro,[1,*] Nuno M. M. Moura,[1,*] Flávio Figueira,[1,3] Rosalina I. Ferreira,[2] Mário M. Q. Simões,[1] José A. S. Cavaleiro,[1] Maria do Amparo F. Faustino,[1,*] Armando J. D. Silvestre,[3] Carmen S. R. Freire,[3] João P. C. Tomé,[4] Shirley Nakagaki,[5] A. Almeida,[2] and M. da Graça P. M. S. Neves[1,*]

1 QOPNA & LAQV-REQUIMTE, Department of Chemistry, University of Aveiro, 3810-193 Aveiro, Portugal.
2 CESAM, Department of Biology, University of Aveiro, 3810-193 Aveiro, Portugal.
3 CICECO, Departamento de Química, Universidade de Aveiro, 3810-193 Aveiro, Portugal.
4 CQE, Departamento de Engenharia Química, Instituto Superior Técnico, Universidade de Lisboa, Av. Rovisco Pais, nº 1, 1049-001 Lisboa, Portugal.
5 Laboratório de Bioinorgânica e Catálise, Departamento de Química, Universidade Federal do Paraná, Curitiba, Brasil.

Contents

^{1}H, ^{13}C and ^{19}F NMR 2
UV-Vis 7
Fluorescence 9
ATR-FTIR 11
PXRD 12
Photostability study 13
Stability of PS-CF after photoinactivation 13

^{1}H, ^{13}C and ^{19}F NMR

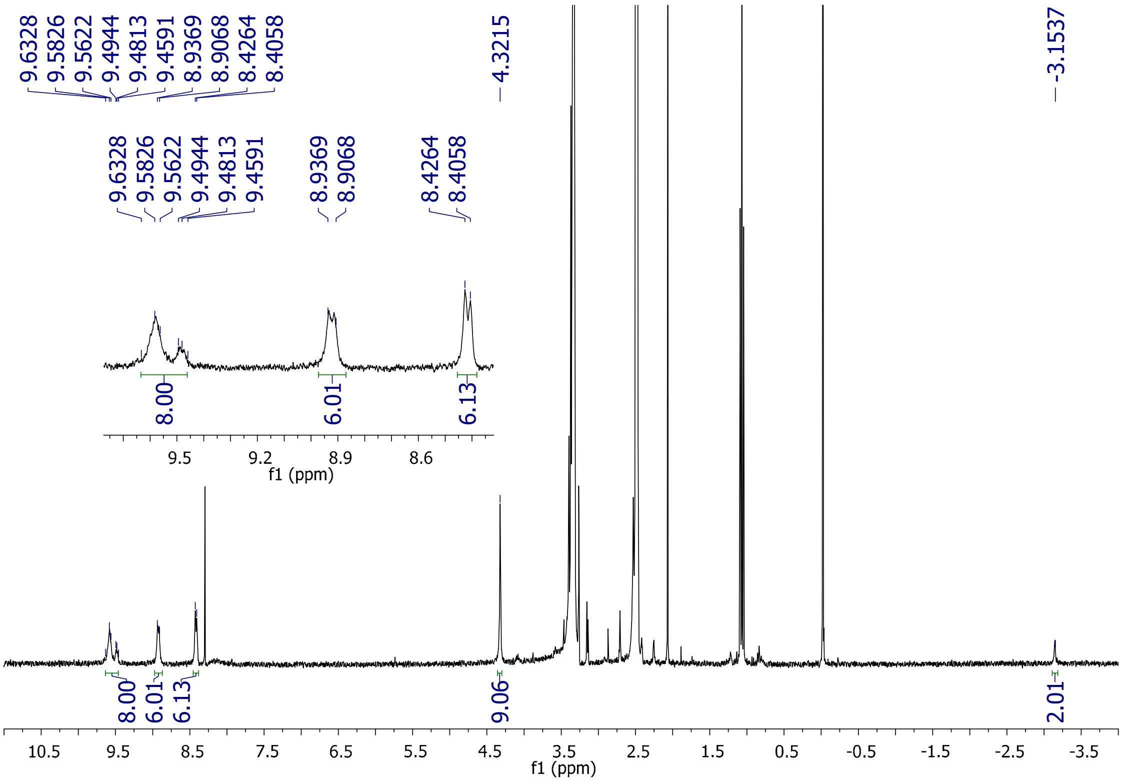

Figure S1. ^{1}H NMR spectrum of compound **P2** in DMSO-d6.

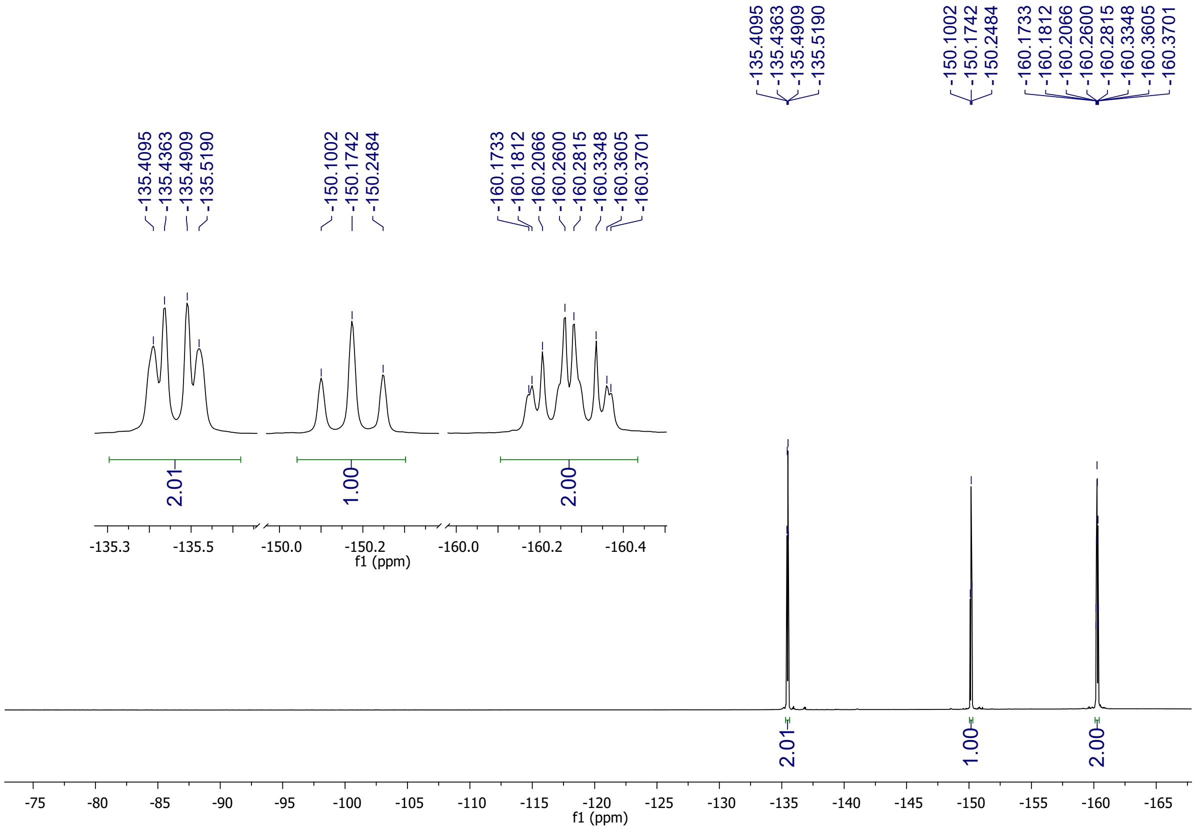

Figure S2. ^{19}F NMR spectrum of compound **P2** in DMSO-d6.

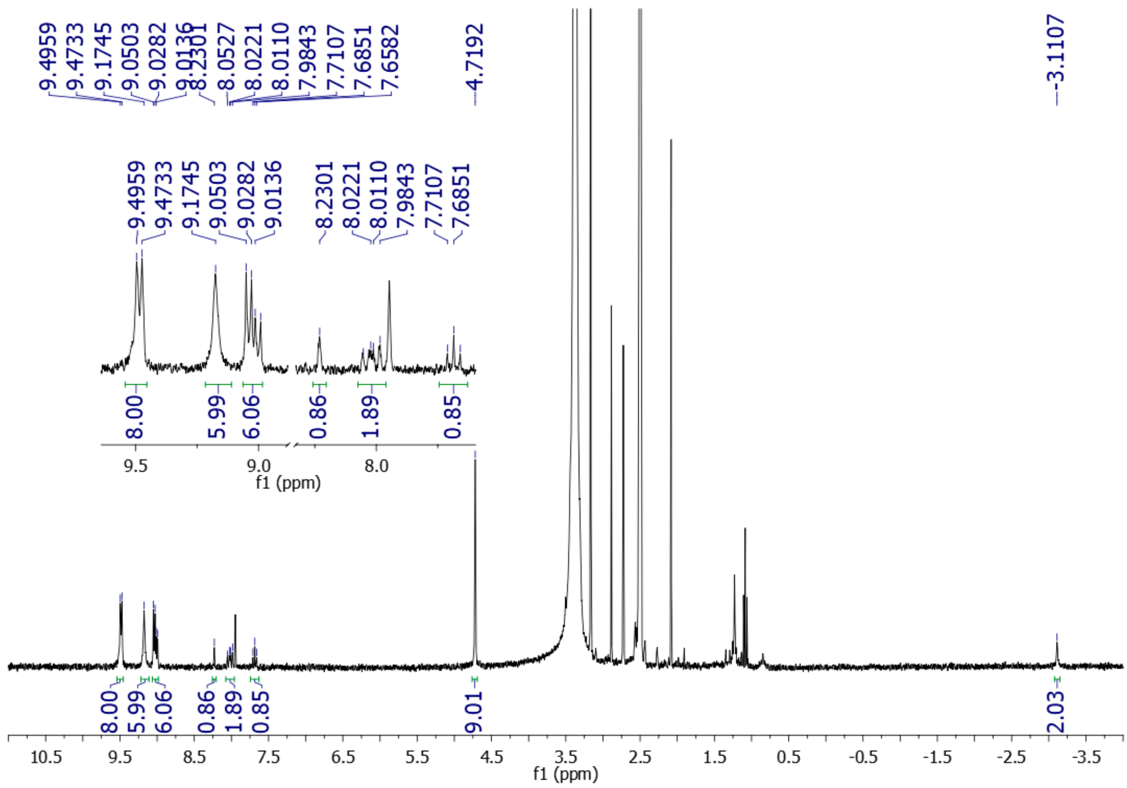

Figure S3. ^{1}H NMR spectrum of compound **P3** in DMSO-d_6.

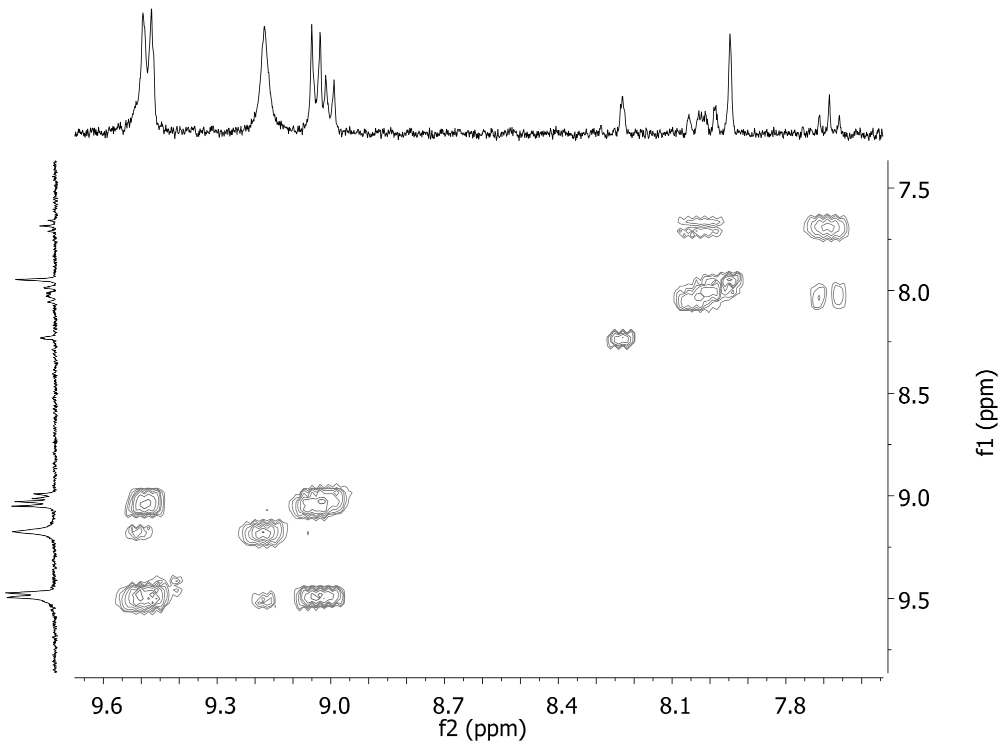

Figure S4. Partial COSY spectrum of compound **P3** in DMSO-d_6.

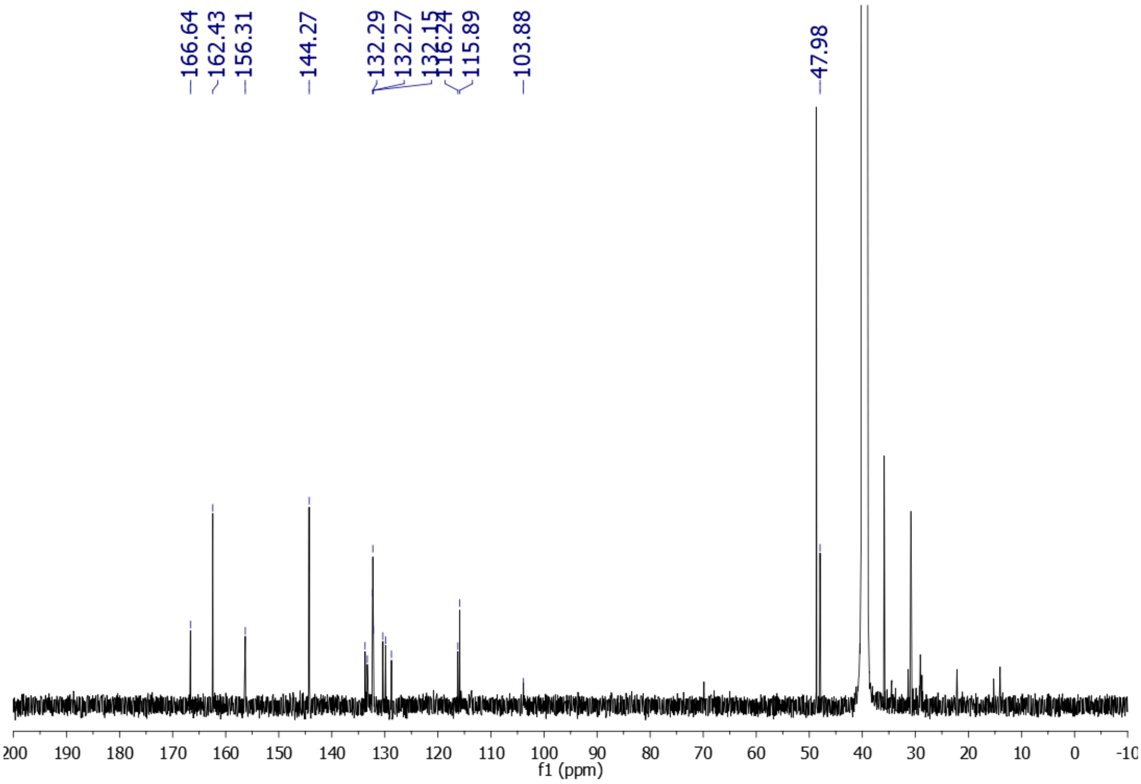

Figure S5. ^{13}C NMR spectrum of compound **P3** in DMSO-d₆.

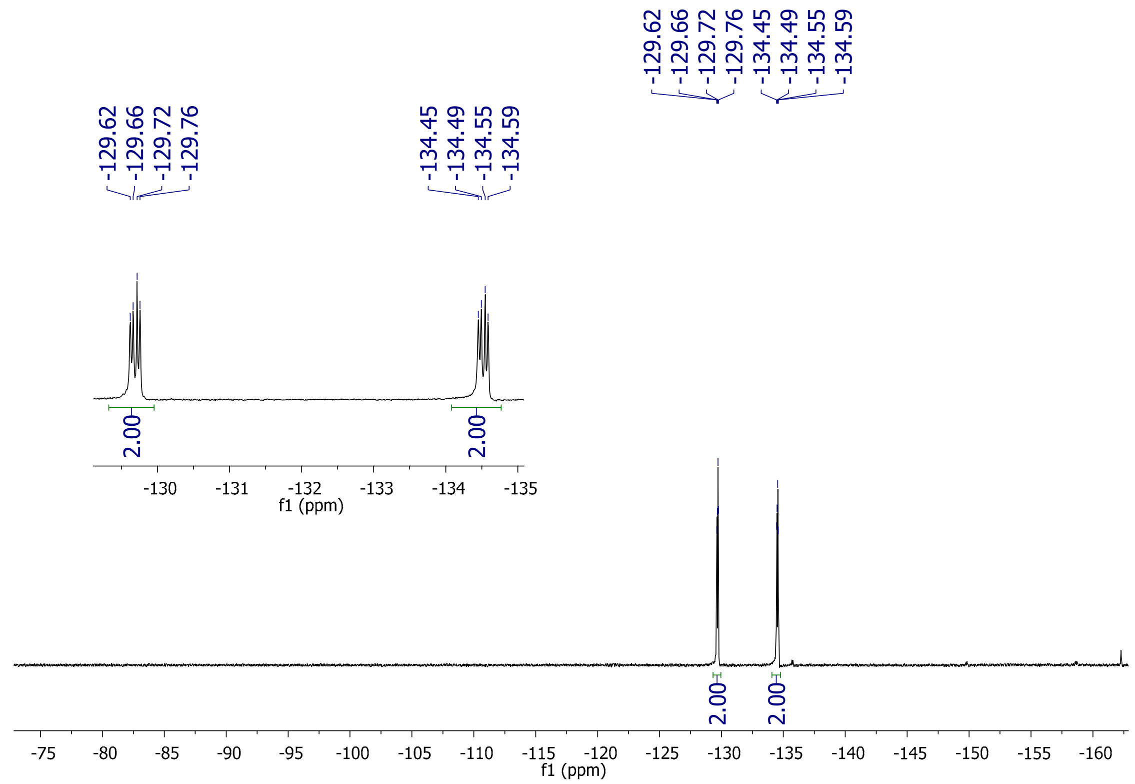

Figure S6. ^{19}F NMR spectrum of compound **P3** in DMSO-d₆.

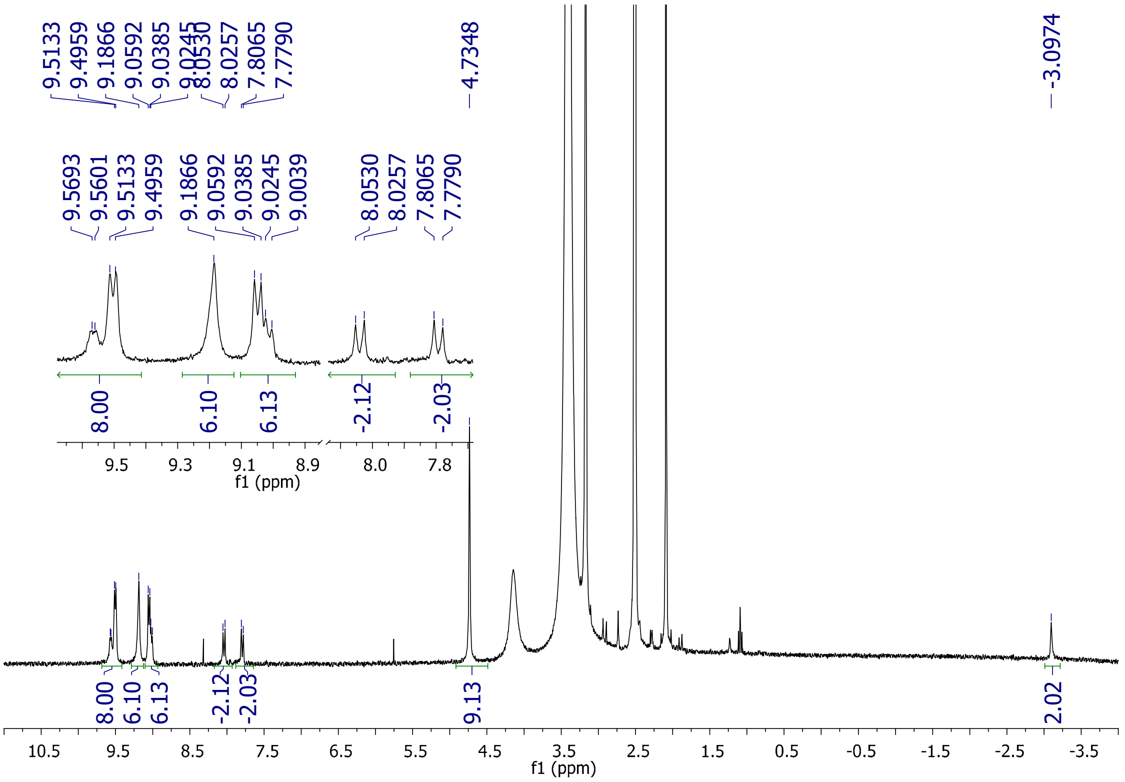

Figure S7. ^{1}H NMR spectrum of compound **P4** in DMSO-d$_6$.

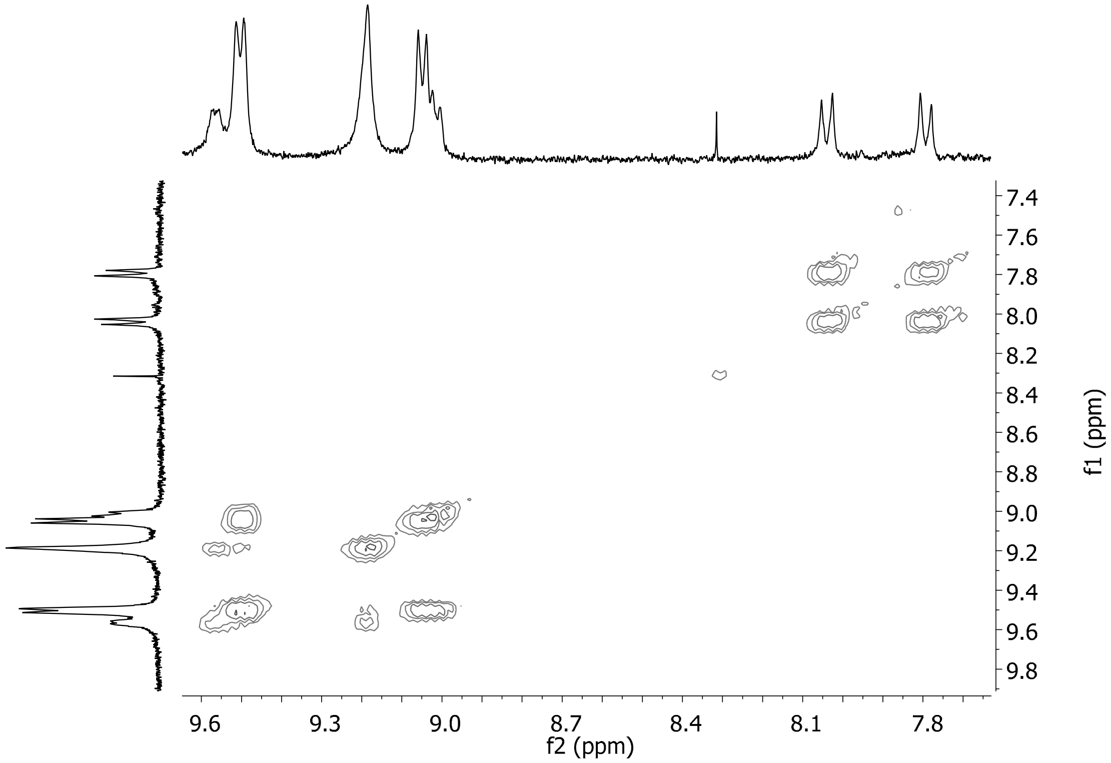

Figure S8. Partial COSY spectrum of compound **P4** in DMSO-d$_6$.

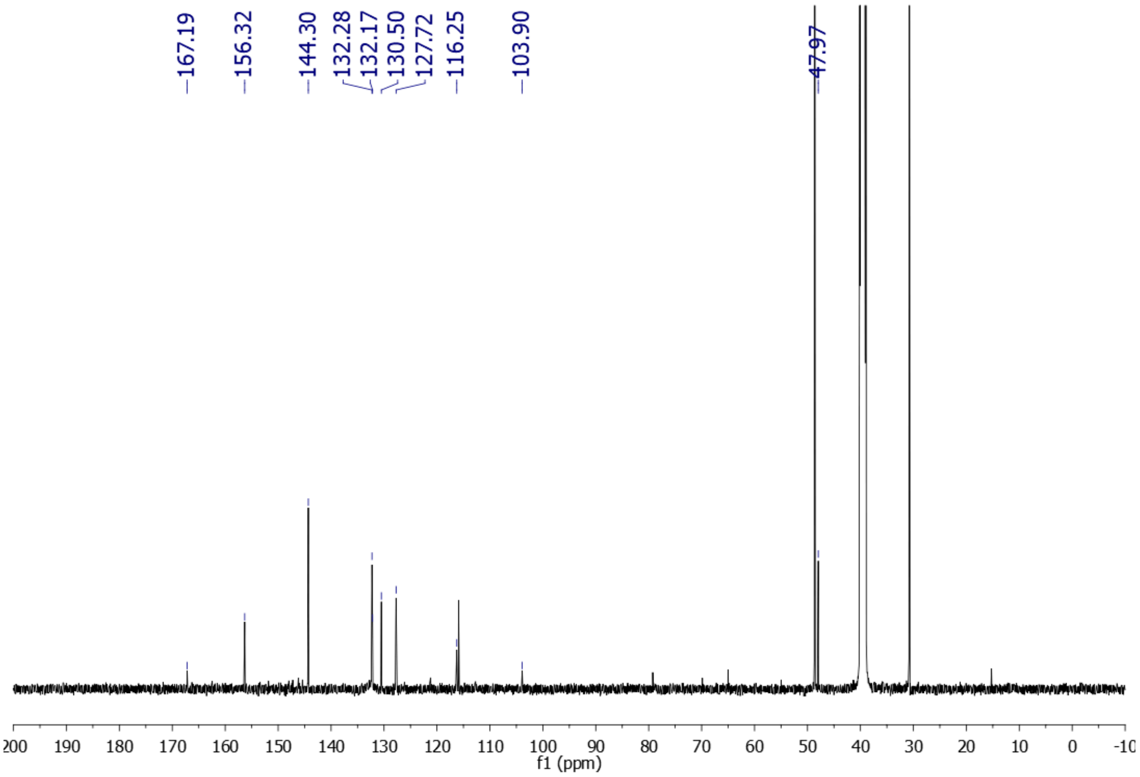

Figure S9. ^{13}C NMR spectrum of compound **P4** in DMSO-d6.

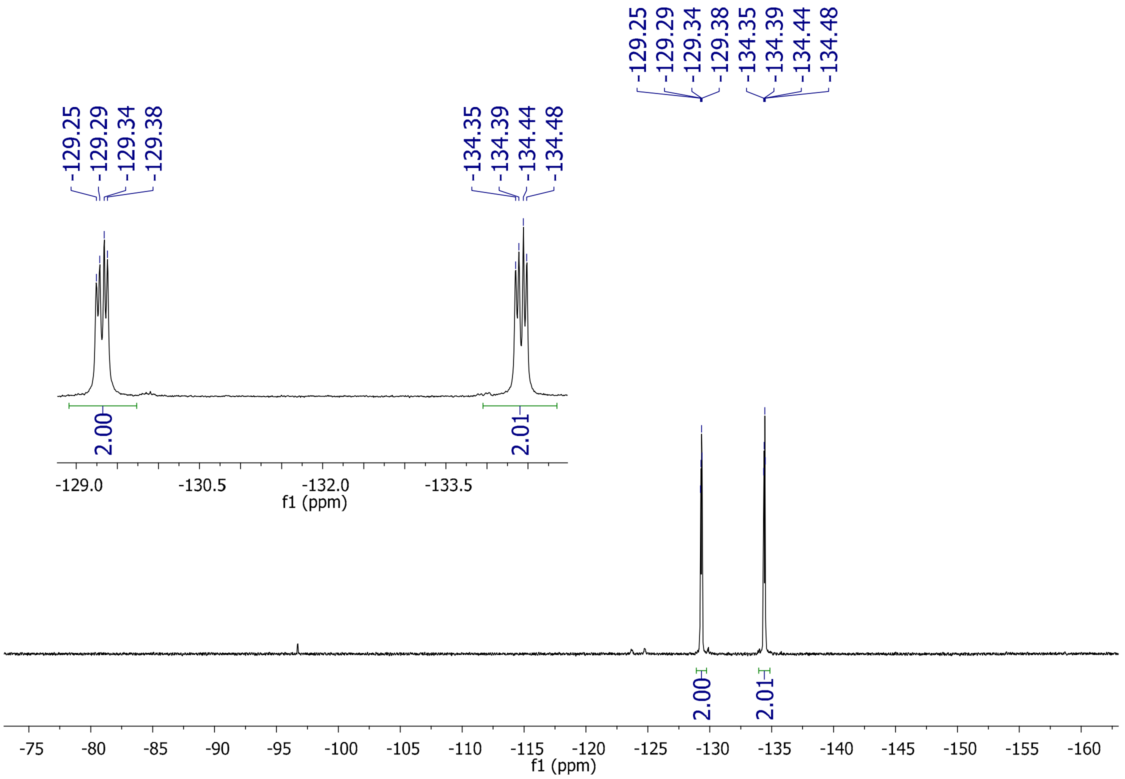

Figure S10. ^{19}F NMR spectrum of compound **P4** in DMSO-d6.

Mass spectrometry

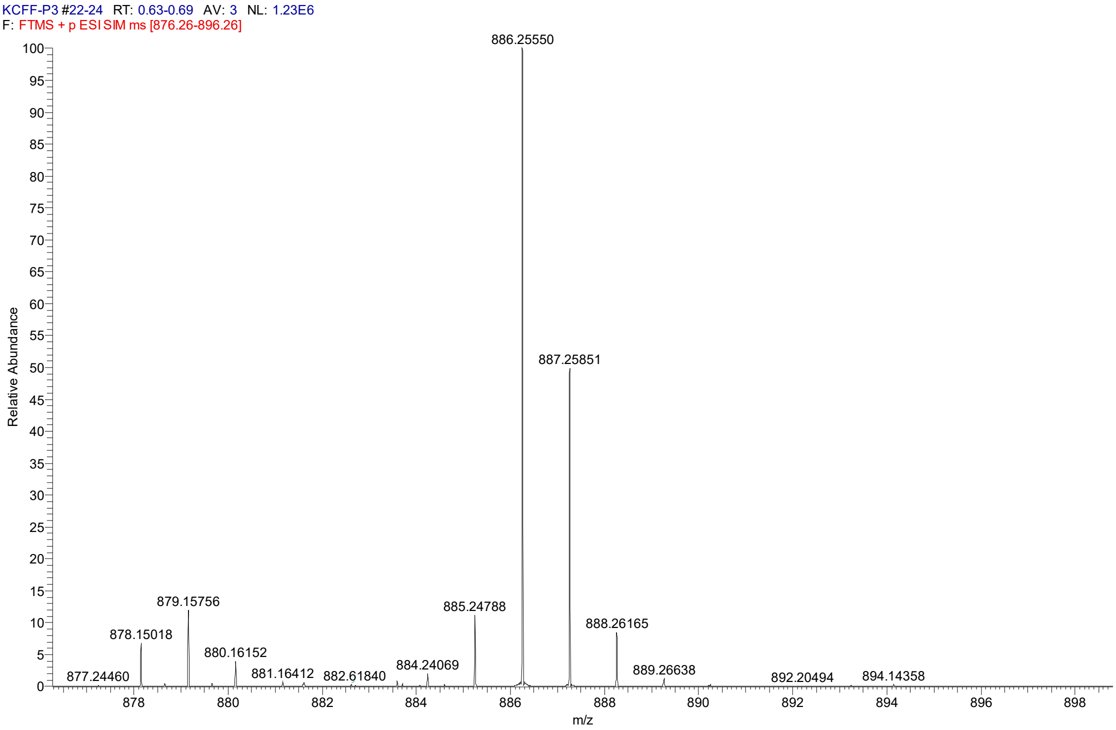

Figure 11. HRMS-ESI(+) spectrum of porphyrin **P3**.

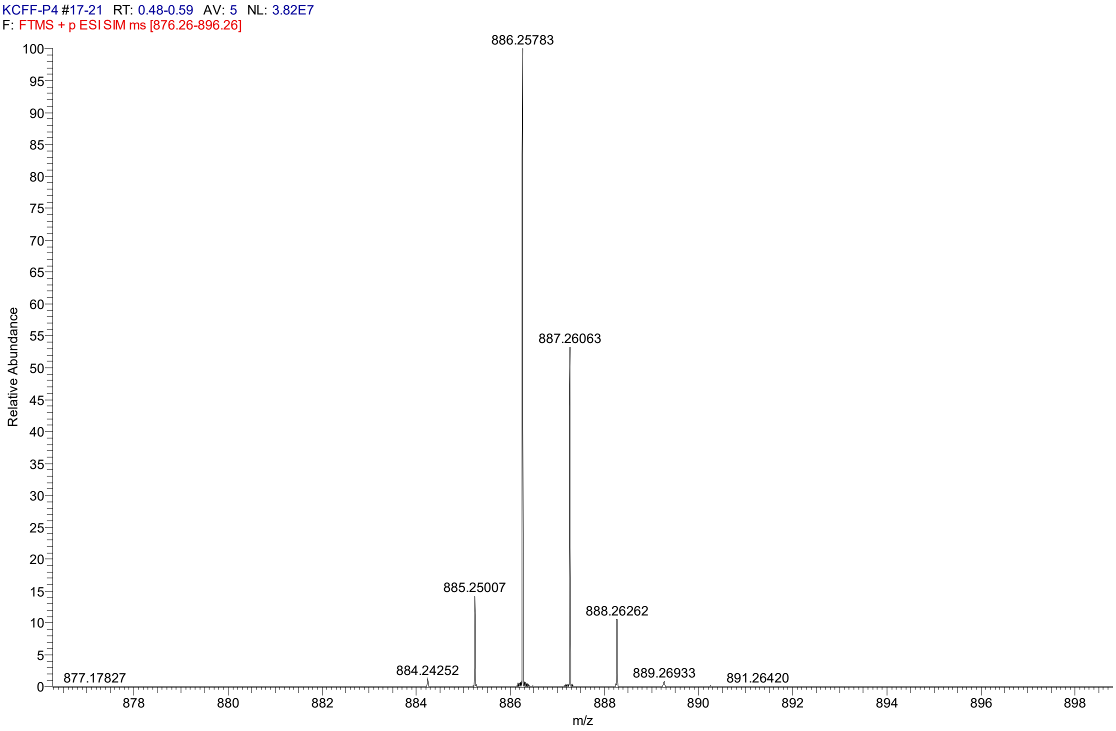

Figure 12. HRMS-ESI(+) spectrum of porphyrin **P4**.

UV-Vis

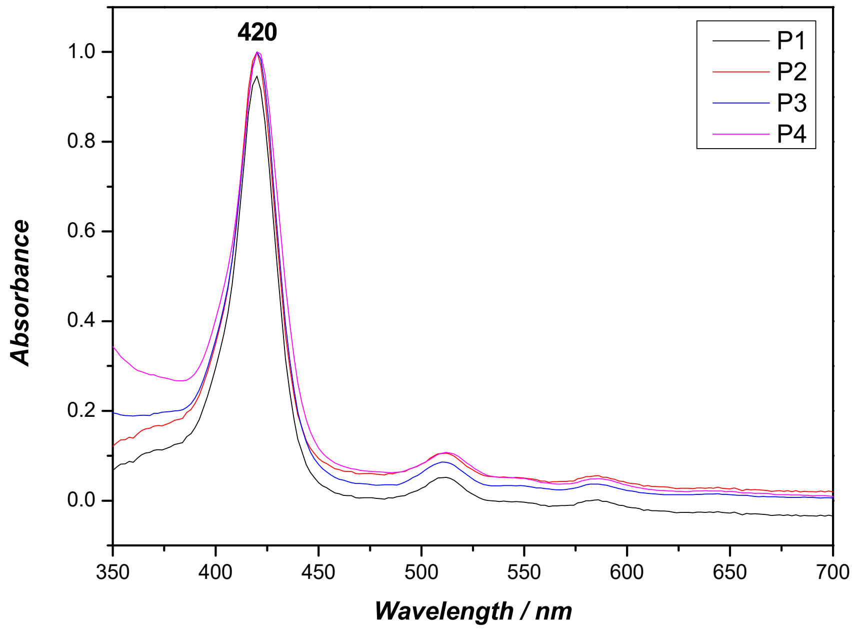

Figure S13. UV-Vis spectra of porphyrins **P1-P4** in DMF. **P1** (black line), **P2** (red line), **P3** (blue line) and **P4** (pink line).

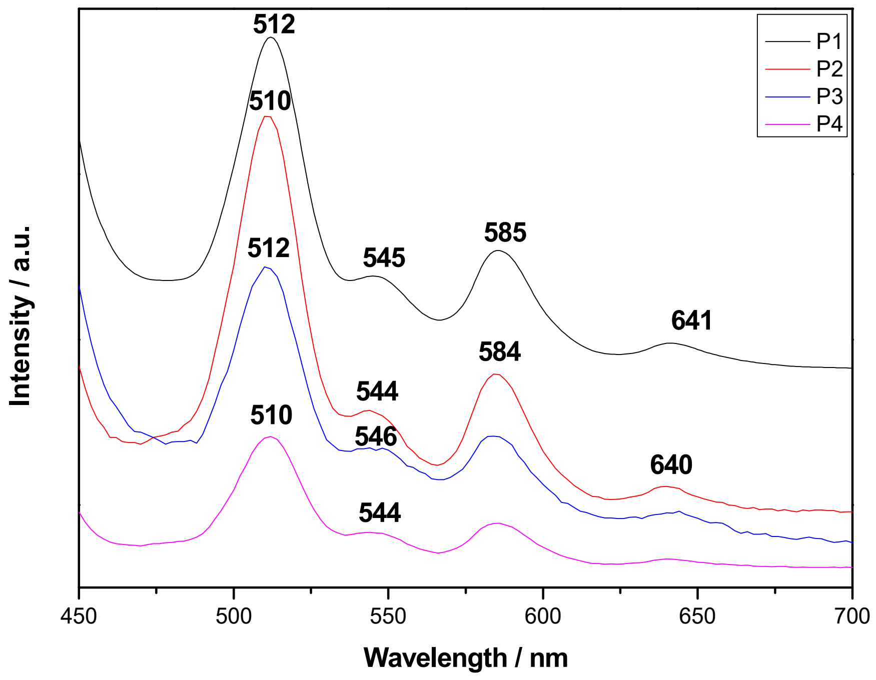

Figure S14. UV-Vis spectra of porphyrins **P1-P4** in DMF (the Q-bands). **P1** (black line), **P2** (red line), **P3** (blue line) and **P4** (pink line).

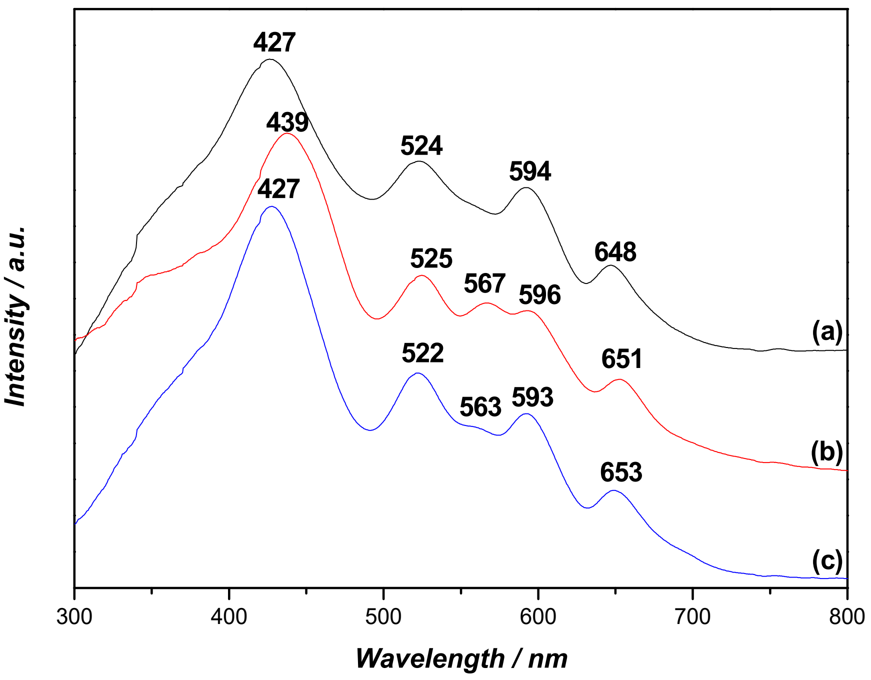

Figure S15. UV-Vis spectra of the solid samples: (a) **P2**, (b) **P3** and (c) **P4**.

Fluorescence

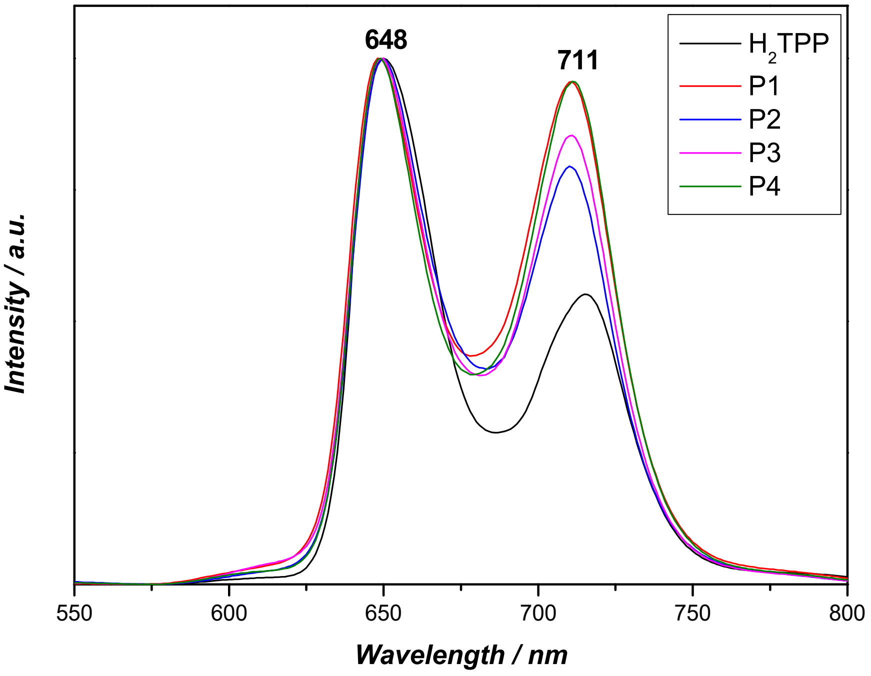

Figure S16. Normalized emission spectra of porphyrins **P1-P4** and **H_2TPP** in DMF: λ excitation at 420 nm. **H_2TPP** (black line), **P1** (red line), **P2** (blue line) **P3** (pink line) and **P4** (green line).

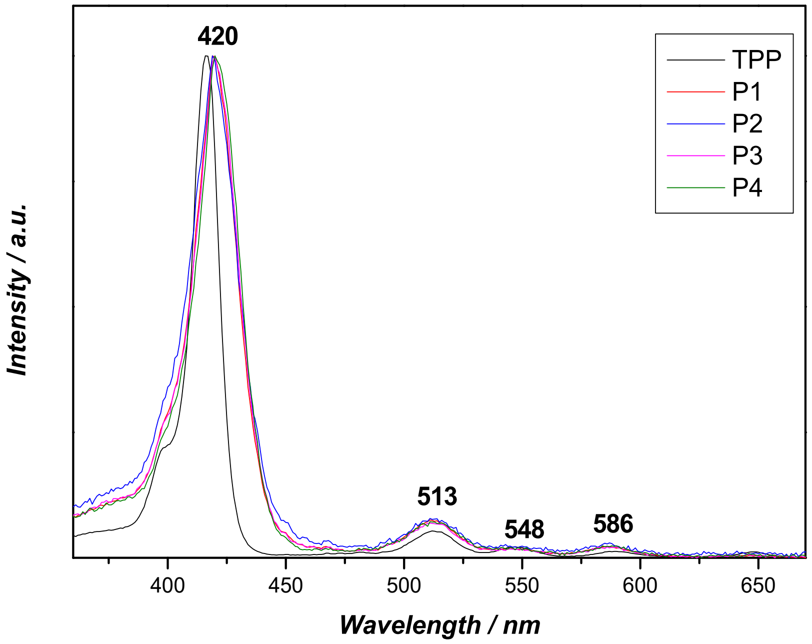

Figure S17. Normalized excitation spectra of porphyrins **P1-P4** and **H_2TPP** in DMF: λ emission at 711 nm. **H_2TPP** (black line), **P1** (red line), **P2** (blue line) **P3** (pink line) and **P4** (green line).

ATR-FTIR

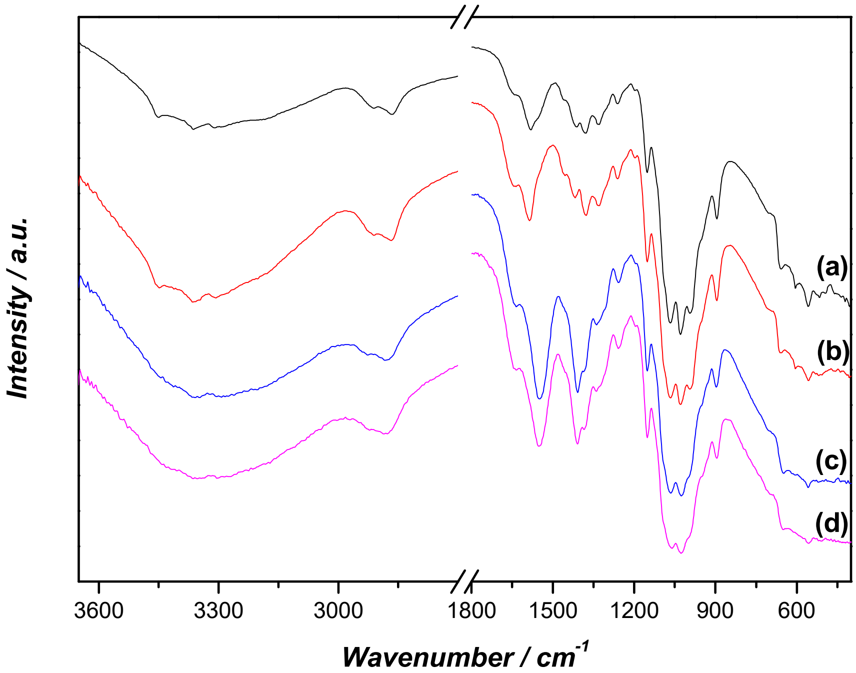

Figure S18. ATR-FTIR spectra of (a) **CF**, (b) **P2-CF**, (c) **P3-CF** and (d) **P4-CF**.

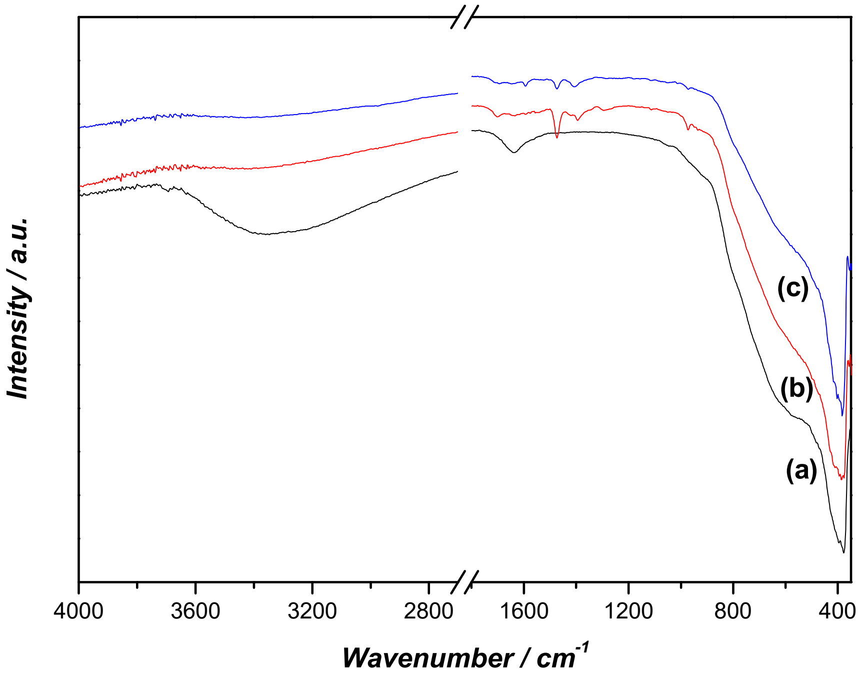

Figure S19. ATR-FTIR spectra of (a) **TiO_2**, (b) **P3-TiO_2** and (c) **P4-TiO_2**.

PXRD

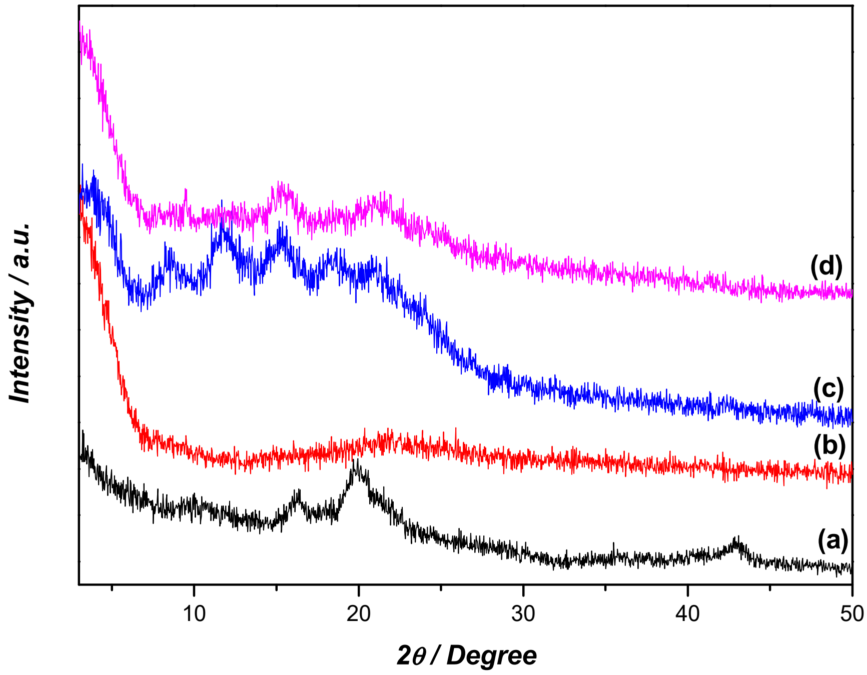

Figure S20. PXRD for (a) **CF**, (b) **P2-CF**, (c) **P3-CF** and (d) **P4-CF**.

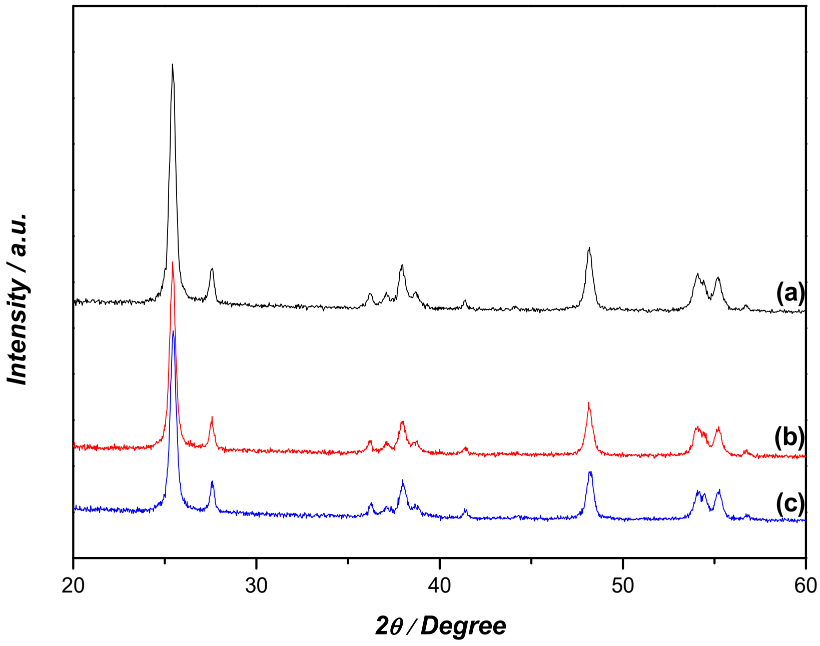

Figure S21. PXRD for (a) $\mathbf{TiO_2}$, (b) $\mathbf{P3\text{-}TiO_2}$ and (c) $\mathbf{P4\text{-}TiO_2}$.

Photostability study

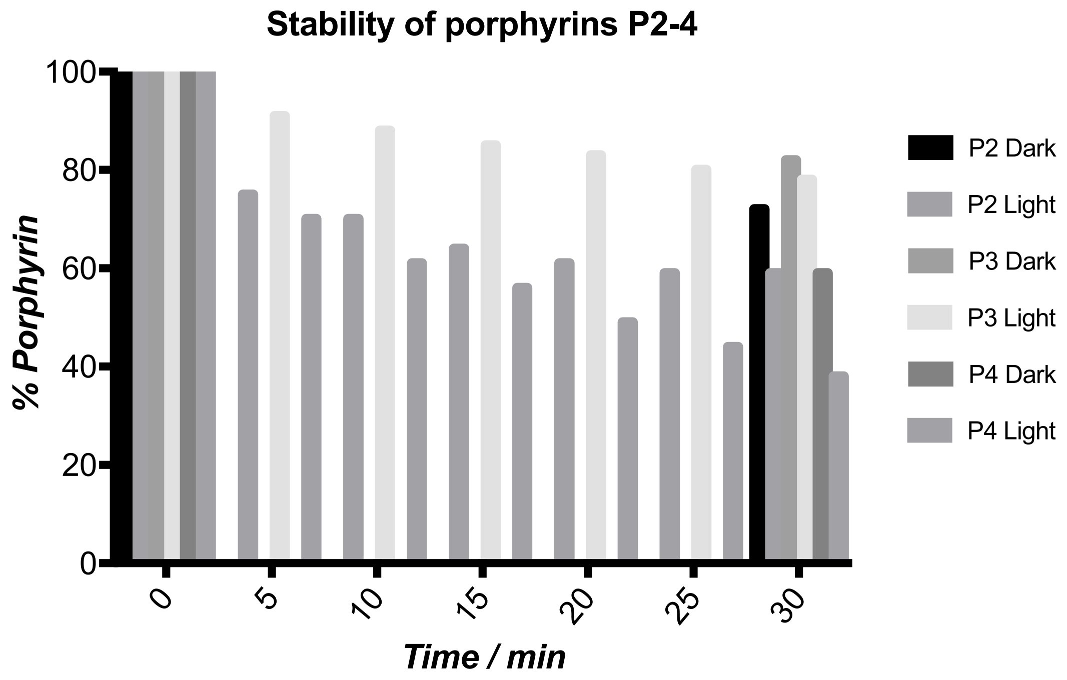

Figure S22. UV-Vis spectrophotometric study of **P2-P4** solution in dark and after irradiation at different times using the same conditions of PDI experiments. The Soret band intensity was monitored over time. The ordinate axis shows the percentage of porphyrin in the PBS solution.

Stability of PS-CF after photoinactivation

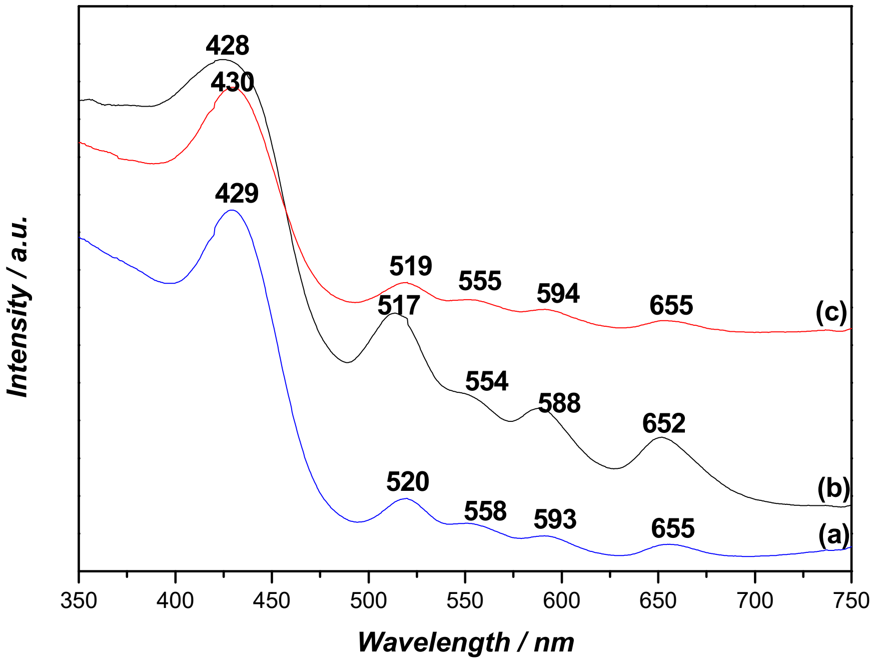

Figure S23. UV-Vis spectra of PS-CF after photoinactivation. **P2-CF** after first use (black line), **P3-CF** after first use (red line) and **P4-CF** after first use (blue line).
